# Structural Features Governing the Metabolic Stability of Tetraethyl-Substituted Nitroxides in Rat Liver Microsomes

**DOI:** 10.3390/antiox12020402

**Published:** 2023-02-07

**Authors:** Aleksandra Rančić, Nikola Babić, Maylis Orio, Fabienne Peyrot

**Affiliations:** 1 Laboratoire de Chimie et de Biochimie Pharmacologiques et Toxicologiques, Université Paris Cité, CNRS, F-75006 Paris, France; 2 iSm2, Aix-Marseille University, CNRS, Centrale Marseille, F-13397 Marseille, France; 3 Institut National Supérieur du Professorat et de l’Education (INSPE) de l’Académie de Paris, Sorbonne Université, F-75016 Paris, France

**Keywords:** aminoxyl radical, nitroxyl radical, spin probe, electron paramagnetic resonance (EPR), electron spin resonance (ESR), microsomes, cytochrome P450

## Abstract

Nitroxides are potent tools for studying biological systems by electron paramagnetic resonance (EPR). Whatever the application, a certain stability is necessary for successful detection. Since conventional tetramethyl-substituted cyclic nitroxides have insufficient in vivo stability, efforts have recently been made to synthesize more stable, tetraethyl-substituted nitroxides. In our previous study on piperidine nitroxides, the introduction of steric hindrance around the nitroxide moiety successfully increased the resistance to reduction into hydroxylamine. However, it also rendered the carbon backbone susceptible to modifications by xenobiotic metabolism due to increased lipophilicity. Here, we focus on a new series of three nitroxide candidates with tetraethyl substitution, namely with pyrrolidine, pyrroline, and isoindoline cores, to identify which structural features afford increased stability for future probe design and application in in vivo EPR imaging. In the presence of rat liver microsomes, pyrrolidine and pyrroline tetraethyl nitroxides exhibited a higher stability than isoindoline nitroxide, which was studied in detail by HPLC-HRMS. Multiple metabolites suggest that the aerobic transformation of tetraethyl isoindoline nitroxide is initiated by hydrogen abstraction by P450-Fe^V^ = O from one of the ethyl groups, followed by rearrangement and further modifications by cytochrome P450, as supported by DFT calculations. Under anaerobic conditions, only reduction by rat liver microsomes was observed with involvement of P450-Fe^II^.

## 1. Introduction

Aminoxyl radicals or nitroxides are paramagnetic compounds of minimal toxicity [1,2]. Therefore, they are convenient tools in chemistry and biology [3,4] as redox [5,6,7,8,9], oxygen [10], and pH-sensitive probes [11,12] or spin labels of biological molecules [13] in association with electron paramagnetic resonance (EPR) spectroscopy and imaging. They have also been used as MRI contrast agents [14,15,16] and behave as antioxidants [17] as they mimic superoxide dismutase (SOD) activity in biological environments [18,19]. Some of these applications require the persistence of the radical character of nitroxide. In contrast, others, such as redox EPR imaging, make use of its propensity to take part in redox reactions and to be reduced to hydroxylamine or oxidized to oxoammonium cation, both of which are diamagnetic and undetectable by EPR. The rate of variation of the EPR signal intensity of nitroxide probes can provide information about the redox status of the system, assuming only redox processes are involved in signal decay [20]. Yet, other events in the probe pharmacokinetics could play a role. For instance, efflux has been shown to interfere with redox status measurements with 3-hydroxymethyl-2,2,5,5-tetramethylpyrrolidine-1-oxyl [21].

The development of biological EPR applications is restricted by the limitations of current probes, which has triggered intensive research in probe design. Cyclic nitroxides are very versatile platforms as they can have different cores, substituents and functional groups. Their reactivity, their solubility and spectral properties highly depend on their structure.

Ascorbate is primarily responsible for converting hydrophilic nitroxides to hydroxylamines in cells [22]. The resistance of nitroxides to reduction by ascorbate is thus an important property to be studied before using them in biological samples or in vivo [23]. Historical studies focused on tetramethyl-substituted structures have shown that the reduction rate of cyclic nitroxides depends on the ring size [24,25]. Substitution and steric shielding of the nitroxides, and more recently the relative configuration of substituents on the ring [26], have also been shown to significantly affect the protection of the nitroxides versus reduction by small bioreductants present in the cytosol of cells. Tetraethyl-substituted nitroxides are consequently increasingly being developed for use in biochemical and biological studies [23,27,28,29,30,31,32].

When lipophilicity increases, however, the high resistance of the nitroxide to reduction leads to an increased time of residence within the cell and, therefore, interaction with metabolic enzymes may no longer be negligible, especially in organs with a high metabolic potential such as the liver. Thus, it is essential to decrypt the metabolic pathways of different nitroxide probes before they are used in animals [33]. An established in vitro model to study metabolic stability is rat liver microsomes (RLM), fragments of endoplasmic reticulum obtained by centrifugation of homogenized tissue that contain all the membrane-associated enzymes involved in xenobiotic metabolisms, such as cytochromes P450 and their reductases [34]. Several groups have thoroughly investigated the metabolic stability of tetramethyl-substituted nitroxides using subcellular fraction models and whole cells. They highlighted the sensitivity to oxygen, the importance of mitochondrial electron-transport-chain enzyme, the hexose phosphate shunt soluble enzymes, cytochrome-P450 reductase and other NAD(P)H-dependent reductases, but not of P450 itself, in the reduction of nitroxides to hydroxylamines [1,35,36,37,38,39,40]. More recently, our study of tetraethyl-substituted piperidine nitroxides TEEPONE (2,2,6,6-tetraethyl-4-oxo-piperidinyloxyl) and TEEPOL (2,2,6,6-tetraethyl-4-hydroxy(piperidine-1-yloxyl)) in aerobic incubations with RLM supplemented with NADPH by EPR and high-performance liquid chromatography coupled to high-resolution mass spectrometry (HPLC-HRMS) demonstrated that they were not reduced significantly to the corresponding hydroxylamines [41]. Instead, P450 catalyzed the oxidation of the hydrocarbon backbone at different sites. By contrast, TEEPOL reacted more slowly, and the piperidine nitroxide moiety was preserved in the products. The observed results helped us to rationalize why in vitro [42,43] and in vivo experiments [44] using TEEPOL and TEEPONE have shown that, even though both nitroxides exhibited better stability toward reduction compared to their tetramethyl equivalents, the latter appeared more unstable in vivo [44]. As a consequence, piperidine nitroxide probes with a sp^2^ carbon in general may be quickly degraded in organs containing high P450 content such as the liver and kidneys.

**Figure 1:**
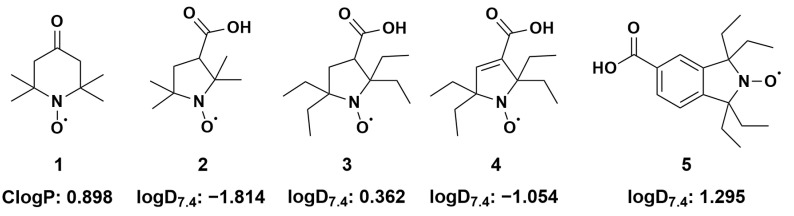
Structural formulas of investigated nitroxides and corresponding ClogP (calculated n-octanol/water partition coefficient) and $logD_{7.4}$ (distribution coefficients at pH 7.4). (**1**) 2,2,6,6-tetramethyl-4-oxo(piperidine-1-yloxyl) radical (TEMPONE) (**2**) 3-carboxy-2,2,5,5-tetramethyl-1-pyrrolidinyloxyl; (**3**) 3-carboxy-2,2,5,5-tetraethyl-1-pyrrolidinyloxyl; (**4**) 3-carboxy-2,2,5,5-tetrethyl-pyrrolinyloxyl; (**5**) 5-carboxy-1,1,3,3-tetraethyl-2-isoindolinyloxyl; Details of calculations are given in the Material and Methods section and in Supplementary Table S1.

The lack of metabolic data on tetraethyl-substituted nitroxides with pyrrolidine, pyrroline and isoindoline rings motivated us to set up the present study. Even though piperidine and pyrrolidine nitroxides are the most commonly used, isoindoline nitroxides have certain advantages. The aromatic moiety in the latter makes them relatively rigid and inert to free radical attack, except for the combination of alkyl radicals at the nitroxide function [45]. In addition, EPR linewidths for isoindoline and pyrroline nitroxides are often narrower, resulting in a higher accuracy in EPR oximetry and imaging, as a resolution is inversely proportional to linewidth [35,46,47]. Here we compared the stability of five nitroxides with different cores (piperidine, pyrrolidine, pyrroline and isoindoline) and substitutions at the α-position to nitroxide (tetramethyl- and tetraethyl-), Figure 1, and assessed their metabolic stability in rat liver microsomes enriched in NADPH by EPR spectroscopy. Experiments were performed under both aerobic and anaerobic conditions. Special attention was devoted to nitroxide **5** with isoindoline core, the metabolism of which was further investigated using HPLC-HRMS and density functional theory (DFT).

## 2. Material and Methods

### 2.1. Reagents

Nitroxide **1** (2,2,6,6-tetramethyl-4-oxo-1-piperidinyloxyl; TEMPONE), diphenyliodinium chloride (DPI), clotrimazole (Clo), (+)-sodium L-ascorbate, deuterated dimethyl sulfoxide (DMSO) and diethylenetriaminepentaacetic acid (DTPA) were purchased from Sigma-Aldrich, potassium ferricyanide from Merck and NADPH tetra-sodium salt from Roche. Nitroxides **2–5** (3-carboxy-2,2,5,5-tetramethyl-1-pyrrolidinyloxyl; 3-carboxy-2,2,5,5-tetraethyl-1-pyrrolidinyloxyl; 3-carboxy-2,2,5,5-tetrethyl-pyrrolinyloxyl; 5-carboxy-1,1,3,3-tetraethyl-2-isoindolinyloxyl) were synthesized according to previously established protocols [30,48,49,50]. The identification and purity of nitroxides **2–5** were verified using HPLC-HRMS (see characterization of compound **5** in the Supplementary Material, Figures S1 and S2 [51]). Potassium phosphate buffer (0.1 M, pH 7.4) was prepared using ultrapure water. The other chemicals and solvents were of the highest quality and commercially available. 

### 2.2. Estimation of Lipophilicity

The calculated n-octanol/water partition coefficients (ClogP) for compounds **1** to **5** were obtained from Chemdraw Professional 21.0.0 software (Perkin Elmer Informatics), together with pKa values for compounds **2** to **5**. The distribution coefficients at pH 7.4 ($logD_{7.4}$), which are corrected markers of lipophilicity for ionizable compounds, were derived using the approximation proposed by Scherrer et al. [52] for acids:

(1) $$logD_{7.4}=logP+pKa-pH$$

### 2.3. Rat Liver Microsome Preparation

Male Sprague–Dawley rats (200–250 g, Charles River, L’Arbresle, France) were adapted in the laboratory for 7 days with free access to laboratory chow and water, and treated with phenobarbital (20 mg·kg^−1^, in corn oil, intraperitoneally for 4 days) before being killed. Rat liver microsomes (RLM) were prepared by differential centrifugation as previously described [53].

The RLM batch was stored at −80 °C and divided into several aliquots before use. During the experiments, the aliquots were stored in ice while the residues were discarded to avoid multiple freeze–thaw cycles that could affect enzyme activity. 

Protein concentrations were determined by the Bradford method [54] using bovine serum albumin (BSA) as a standard and were 25 ± 2 mg·ml^−1^. Cytochrome c reductase activity and cytochrome P450 concentration were determined by UV-VIS spectroscopy according to the methods of Vermilion et al. [55] and Omura et al. [56]. The P450 concentration in microsomes was 100 μM. The cytochrome c reductase activity corresponded to 216 ± 6 nmol·min^−1^·mg^−1^.

### 2.4. Reactions of Nitroxides with Ascorbic Acid Monitored by EPR Spectroscopy

EPR spectra of mixtures of nitroxides and sodium ascorbate in potassium phosphate buffer (0.1 M, pH 7.4, containing 1 mM DTPA) were recorded at 21 °C using an Elexsys E500 spectrometer (Bruker, Wissembourg, France) operating in X-band (9.8 GHz) and equipped with a high sensitivity SHQ cavity. A 4-bore AquaX quartz cell (Bruker) inserted into the cavity was connected to a Bio-Logic MPS-51 stop-flow apparatus with three syringes (Bio-Logic, Claix, France) controlled by the Bio-Logic MPS software. Field-time 2D acquisitions were performed with the following acquisition parameters: modulation frequency, 100 kHz; modulation amplitude, 0.10 mT for compounds **1** and **2**, 0.30 mT for compound **3**, 0.15 mT for compound **4**, and 0.14 mT for compound **5**; time constant, 20.48 ms; conversion time, 20.49 ms; center field, 350.3 mT; sweep width, 5.0 mT; sweep time, 10.49 s; microwave power, 10 mW. Data acquisition and processing were performed using Bruker Xepr software. The initial second-order reaction rate constant of the reaction of nitroxides with ascorbate, k_0_ values was derived from experimental results as previously described [57,58].

### 2.5. Reactions of Nitroxides with RLM Monitored by EPR Spectroscopy

Nitroxide stock solutions were prepared in DMSO at a concentration of 50 mM. For experiments performed under aerobic conditions, the samples under study were assembled in potassium phosphate buffer (0.1 M, pH 7.4, containing 1 mM DTPA) by diluting 50 mM nitroxide stock solutions to the concentration of 100 μM, adding RLM to the P450 concentration of 1 μM, and finally adding 1 mM NADPH to initiate the reaction. After vortexing, samples were transferred into a gas-permeable PTFE tube (Extruded Sub-Lite-Wall, inner diameter 0.635 mm, wall thickness 0.051 mm; Zeus Industrial Products Ltd., Ireland), which was folded into a V-shape in a 4-mm quartz tube and then placed in the EPR SHQ high-sensitivity cavity. 

For measurements under anaerobic conditions, nitroxides and RLM diluted in degassed, nitrogen-saturated potassium phosphate buffer (0.1 M, pH 7.4, containing 1 mM DTPA) to the same concentration as in the aerobic study, were degassed separately from 10 mM NADPH solution for 10 min. Addition of NADPH to a final concentration of 1 mM started the reaction. Samples were mixed and transferred to glass microcapillary pipettes (50 μL, Hirschmann) that had been previously purged with N_2_. The pipettes were sealed with CRITOSEAL paste (Leica), placed in a 4-mm quartz tube, and then in the EPR cavity.

Control samples were prepared without NADPH or microsomes, with Clo (25 μM final, stock solution in DMSO) or DPI (100 μM final, stock solution prepared extemporaneously) under both aerobic and anaerobic conditions. A 100 mM aqueous solution of potassium ferricyanide was used for reoxidation. It was added to the mixture of nitroxide, RLM and NADPH in potassium phosphate buffer (0.1 M, pH 7.4, containing 1 mM DTPA) previously incubated for 30 min. The final concentration of this oxidant was 1 mM. 

EPR measurements were performed with the following settings: microwave power, 1 mW; modulation frequency, 100 kHz; modulation amplitude, 0.10 mT for nitroxides **1**, **2**, **4** and **5** or 0.30 mT for nitroxide **3**; receiver gain, 60 dB; time constant, 40.96 ms for nitroxides **1**, **2**, **4** and **5** or 81.92 ms for nitroxide **3**; conversion time, 40.96 ms for nitroxides **1**, **2**, **4** and **5** or 81.32 ms for nitroxide **3**; data points, 1024 for nitroxides **1**, **2**, **4** and **5** or 512 for nitroxide **3**; sweep width, 10 mT for nitroxides **1**, **3** and **4** or 5 mT for nitroxide **2** or 8 mT for nitroxide **5**; sweep time, 41.94 s. EPR kinetics were recorded at 21 °C for 30 min. Data acquisition and processing were performed using Bruker Xepr software. The settings used in recording the superhyperfine structure of nitroxide **5** were: microwave power, 1 mW; modulation frequency, 100 kHz; modulation amplitude, 0.004 mT; receiver gain, 60 dB; time constant, 40.96 ms; conversion time, 40.96 ms; data points, 1024; sweep width, 1 mT; sweep time, 41.94 s.

### 2.6. Calibration and Simulation of EPR Spectra

Quantification of EPR intensities was obtained by using calibration standard solutions of each nitroxide prepared in buffer. Computer simulations of EPR spectra were performed with EasySpin toolbox (garlic function) [59] in Matlab software, version R2016a (Mathworks, Natick, MA, USA).

### 2.7. Statistical Analyses

Statistical significance was estimated by unequal variance t-test. The criterion for significance was *p* < 0.001, *p* < 0.01 or *p* < 0.05.

### 2.8. HPLC-HRMS Study of Isoindoline Nitroxide with RLM

The reaction between nitroxide **5** and sodium ascorbate was first analyzed. For this purpose, nitroxide **5** was degassed in potassium phosphate buffer (0.1 M; pH 7.4) for 10 min. Then, a degassed solution of sodium ascorbate in water was added. The final concentration was 100 μM nitroxide and 800 mM ascorbate. The mixture was incubated under argon for 1 h and examined using HPLC-HRMS.

The study with microsomes was performed under both aerobic and anaerobic conditions. For aerobic samples, nitroxide **5** was mixed with RLM in potassium phosphate buffer (0.1 M, pH 7.4) to a concentration of 300 μM nitroxide and 30 μM P450. Addition of 2 mM NADPH initiated the reaction. A total of 100 μL of the incubation mixture was withdrawn at different times and added to 50 μL of cold acetonitrile to stop the reaction. For the anaerobic experiments, nitroxide **5** was diluted with RLM in degassed, argon-saturated potassium phosphate buffer (0.1 M; pH 7.4) to the same concentrations as for the aerobic studies. The mixture was degassed separately from the 10 mM NADPH solution with argon for 10 min. The degassed NADPH was added to the mixture at a concentration of 2 mM. A total of 100 μL aliquots of the incubation mixture was processed and analyzed under the same conditions as before. In both cases, control experiments were performed without NADPH or nitroxide **5**. All mixtures with acetonitrile were centrifuged at 13 000 rpm with the miniSpin centrifuge (Eppendorf) for 20 min. The supernatants were collected and transferred to HPLC sample vials. 

HPLC-HRMS analyses were performed with a Thermo Exactive_HCD spectrometer (Thermo Scientific, Les Ulis, France) coupled to a Nexera X2 HPLX (Shimadzu, Marne-La-Vallée, France) system using a Phenomenex Kinetex C18 LC column (2.6 μm, 50 × 2.1 mm, 100 Å) with reversed phase thermostated at 40 °C. The gradient was A + B (A = H_2_O + 0.1% HCOOH; B = CH_3_CN + 0.1% HCOOH; at 0.3 mL min^−1^), starting at 10% B and increasing linearly to 100% of B in 30 min, then remaining constant for 5 min, decreasing abruptly to 10% of B, and then remaining constant for 5 min. UV-Vis detector in the range 200–600 nm was performed with a photodiode array (Shimadzu SPD-N20A). Mass spectra were obtained by electrospray ionization in positive (ESI^+^) and negative (ESI^−^) modes under the following conditions: sheath gas, ESI^+^ 4, ESI^−^ 3; auxiliary gas, 0; capillary temperature, 275 °C; and mass range, 150–1000. Data processing was performed using the Thermo Xcalibur program.

### 2.9. Computational Investigations

The ORCA program package was used to perform all theoretical calculations based on DFT [60]. All systems were subjected to full geometry optimization and electronic structure calculations were undertaken using the B3LYP/G functional [61,62] in combination with the 6–31 g* basis sets [63,64,65]. Increased integration grids and tight SCF convergence criteria were used in the calculations. 

Solvent effects were accounted for according to the experimental conditions and water was used as a solvent (ε = 80) within the framework of the conductor-like polarizable continuum model, CPCM [66]. Additional single point calculations using the same level of theory were conducted to predict EPR parameters. Considering the notable influence of solvent (polarity, solute–solvent interactions, hydrogen bond interactions) on the theoretical prediction of hyperfine coupling constants (hfcc) [67,68,69], extra models including one, two and three explicit water molecules in close proximity with nitroxide **5** were built and computed. The results showed that the model with one explicit water molecule was converged and provided the best agreement between experiment and theory (see Supplementary Material). Numerical frequency calculations were performed to confirm that the resulting structures converged to a local minimum on the potential energy surface and resulted in only positive normal vibrations. Molecular orbitals were generated using the orca_plot utility program. Optimized geometries as well as electronic structures were visualized using the program Chemcraft.

## 3. Results

### 3.1. Reactivity of Nitroxides with Ascorbate

According to the literature, the most important reaction of water-soluble nitroxides in the biological medium is the interaction with small water-soluble bioreducing agents such as ascorbate [24]. The rate of reaction of most of the nitroxides selected in the present study (Figure 1) was already known but not that of compound **5**. Therefore, we first investigated the reactivity of the nitroxide series with sodium ascorbate under identical conditions in potassium phosphate buffer (0.1 M, pH 7.4) equilibrated with air using a stopped flow EPR setup. The results in terms of the initial second-order reaction rate constants are summarized in Table 1.

In line with what was already observed in the literature, piperidine nitroxide TEMPONE **1** reduced more rapidly than all other nitroxides under study. Tetraethyl pyrrolidine nitroxide **3** was more stable than its tetramethyl analogue **2** and was confirmed as the most resistant nitroxide in the series. Pyrroline nitroxide **4** was slightly easier to reduce, as already described. Isoindoline nitroxide **5** showed intermediate stability, and its reduction rate was comparable to that of the trityl radical, another important spin probe for biological EPR (k_0_ = 0.016 ± 0.002 M^−1^·s^−1^) [42]. The deprotonation of carboxylic acids in compounds **2** to **5** contributed to the resistance to reduction because an electrostatic interaction developed with the monoanionic ascorbate at a physiological pH. The charge effect was clearly noticeable compared to the published rate for an analogue bearing a tetramethylammonium group in place of the carboxylate in compound **5** (k_0_ = 0.081 ± 0.001 M^−1^·s^−1^) [42].

Ascorbate reduced nitroxides to the corresponding diamagnetic hydroxylamines. We took advantage of this to unambiguously characterize the hydroxylamine derived from nitroxide **5** using HPLC coupled to HRMS. Since steric shielding does not allow rapid reduction of tetraethyl substituted nitroxides by ascorbate, a large amount of sodium ascorbate (800 mM) compared to nitroxide **5** (100 μM) was used. The reaction between nitroxide **5** and sodium ascorbate was carried out under anaerobic conditions to prevent reoxidation of the hydroxylamine back to the nitroxide by oxygen. The total scan UV-Vis and total ion current (TIC) chromatograms obtained after one-hour incubation are shown in Supplementary Figure S4, and the mass spectrum of the hydroxylamine product is shown in Supplementary Figure S5. The latter eluted at 10.9 min, being more polar than nitroxide **5** (RT = 13.6 min). Interestingly, the detection of the hydroxylamine was far more sensitive in ESI^+^ (*m*/*z* calcd. For C_17_H_26_NO_3_ [*MH^+^*] 292.1907; exp. 292.1907) than in ESI^−^ mode, whereas the opposite was true for the nitroxide (Figures S1 and S2). Another important aspect of the hydroxylamine ESI^−^ spectrum was that the dominant species was produced by oxidation in the source in addition to deprotonation (*m*/*z* calcd. for C_17_H_23_NO_3_ [*(M-2H)^−^*] 289.1683; exp. 289.1686).

We then turned to the core of our study in the presence of microsomes from phenobarbital-treated rats with various cofactors and inhibitors. 

### 3.2. Anaerobic Study of Nitroxides Incubated with RLM

Under anaerobic conditions, the kinetics of nitroxides (Figure 1) incubated with RLM enriched in NADPH were monitored by EPR for 30 min. When no oxygen was present in the solution, the EPR spectrum of nitroxide **5** was characterized by a visible superhyperfine structure, as shown in Supplementary Figure S3, in a similar way to other isoindoline nitroxides [46]. This feature was used to verify the degassing process in the experiments. Table 2 summarizes the results presented as initial rates of decrease, which were highly dependent on the nitroxide structure. In the absence of oxygen, nitroxide **2** did not react at all, which contrasts with the previous observations using microsomes from untreated rats [40] and could be due to a difference in expressions and activities of microsomal enzymes. For all other nitroxides, the decay was linear for 15 min at least. As previously observed, the piperidine ring **1** was reduced more rapidly than the other rings [40]. Tetraethyl-pyrrolidine **3** and pyrroline **4** nitroxides did not react significantly in RLM supplemented with NADPH under anaerobic conditions, while nitroxide **5** was transformed at a slow rate. Throughout the reaction time, the shape of the EPR spectra remained constant and was characterized by three lines typical of nitroxides. Therefore, no new paramagnetic product was identified in the course of the reaction. 

All nitroxide reactions were microsomes- and NADPH-dependent, consistent with the fact that an electron donor is required for the P450 catalytic cycle to function. The NADPH-cytochrome P450 reductase transports electrons from NADPH to P450 through flavin mononucleotide (FMN) and flavin adenine dinucleotide (FAD) cofactors [53]. DPI (100 μM final) has been shown to interact with the reduced flavins and inhibit their reactions [72]. Under anaerobic conditions, the addition of this non-specific inhibitor delayed the evolution of TEMPONE **1** and completely stopped the reaction of other nitroxides, demonstrating either the direct or indirect involvement of the reductase. The same effect was obtained when the incubation was treated with Clo (25 μM final), which is a heme-Fe^III^ ligand [73], showing, for the first time to our knowledge, that the reaction between nitroxides and P450 involves the heme active site. By adding potassium ferricyanide (1 mM final) to the whole system after 30 min of incubation, we were able to regenerate the signals from TEMPONE **1** (37%) and nitroxide **5** (30%). Reoxidation was only partial in both cases, indicating that hydroxylamine was not the only product of the reaction. Moreover, reoxidation of nitroxide **5** was possible only upon the simultaneous addition of Clo (25 μM final), which stopped the reaction of P450. Since the change in signal intensity was negligible in the case of nitroxides **3** and **4**, their reoxidation was hardly detectable.

**Figure 2:**
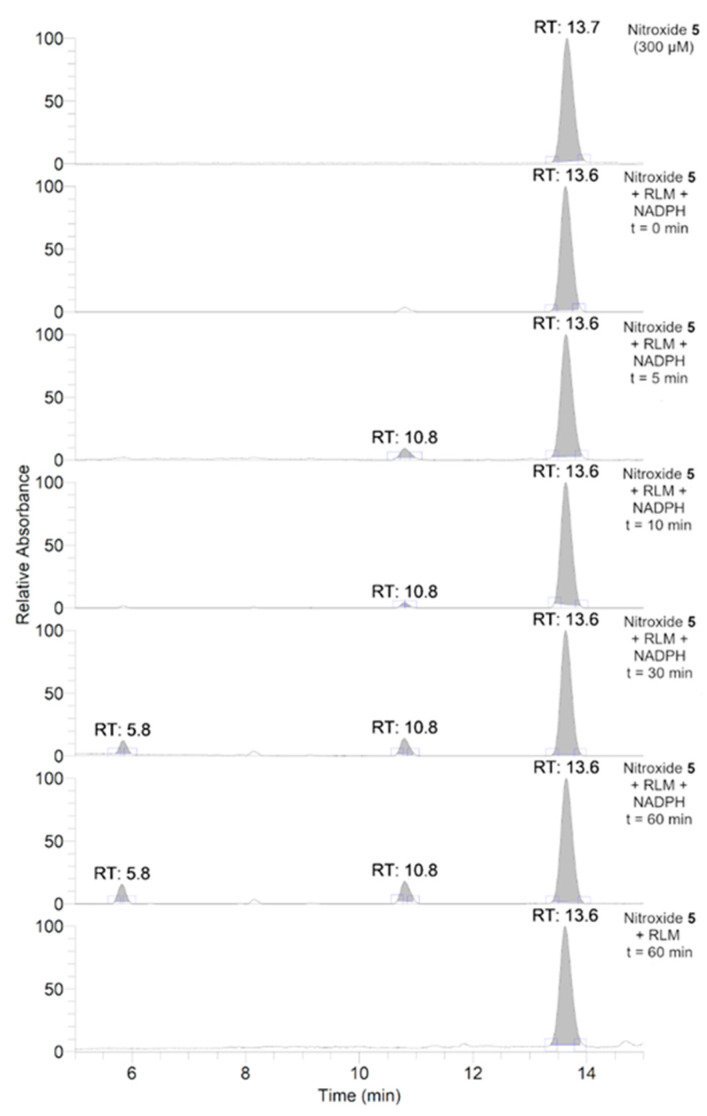
Total scan UV-Vis chromatograms of the incubations of nitroxide **5** (300 μM) in RLM (7.8 mg·mL^−1^ protein, 30 μM P450) with or without NADPH (2 mM) in potassium phosphate buffer (100 mM, pH 7.4) upon anaerobic conditions. Samples were prepared and analyzed as described in the experimental section.

**Figure 3:**
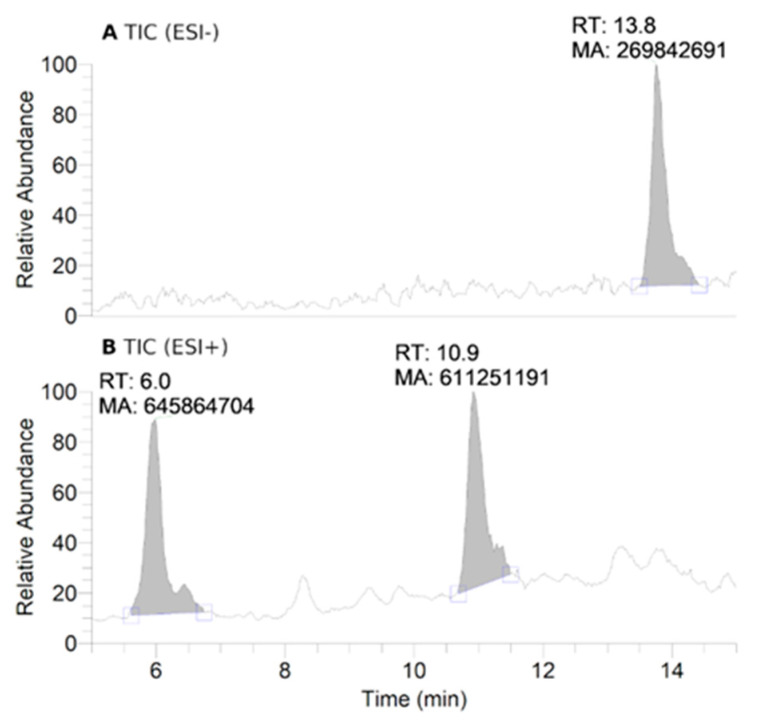

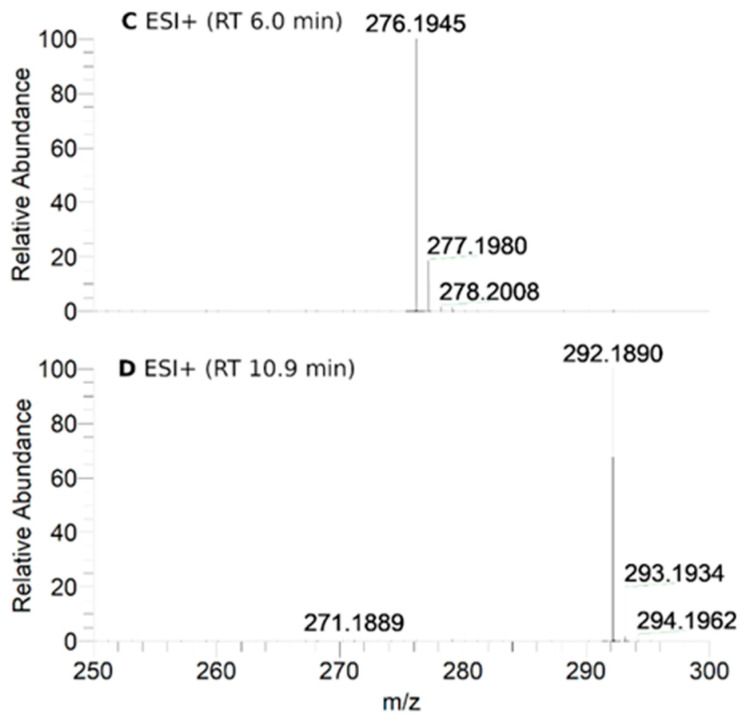
HPLC-HRMS study of nitroxide **5** (300 μM) incubated with RLM (7.8 mg·mL^−1^ protein, 30 μM P450) supplemented with NADPH (2 mM) in potassium phosphate buffer (0.1 M, pH 7.4) upon anaerobic conditions after 60 min: total ion current (TIC) chromatograms of the principal ions in negative (ESI^−^) (**A**) and positive electrospray ionization (ESI^+^) mode (**B**); ESI^+^ HRMS spectra of the principal products at retention times (RT) = 6.0 (**C**) and 10.9 min (**D**). Peaks in TIC chromatograms are observed with a delay of 0.2 min compared to the UV-Vis detection.

To further investigate the transformations of nitroxide **5**, we performed a HPLC-HRMS study. Nitroxide **5** was incubated with RLM supplemented with NADPH for different time periods (up to 60 min). The total scan UV-Vis chromatograms are shown in Figure 2. Nitroxide **5** eluted at 13.6 min and, when incubated with RLM and NADPH without oxygen, it was accompanied by two more small peaks at 5.8 and 10.8 min, the latter appearing at earlier incubation times. The TIC chromatograms and the mass spectra corresponding to the main products are shown in Figure 3. The product initially formed and eluting at 10.8 min was consistent with the hydroxylamine (*m*/*z* calcd. for C_17_H_26_NO_3_ [(*M+H*)*^+^*] 292.1907; exp. 292.1890), while the latter product eluting at 5.8 min corresponded to the amine (*m*/*z* calcd. for C_17_H_26_NO_2_ [(*M+H*)*^+^*] 276.1958; exp. 276.1945).

**Figure 4:**
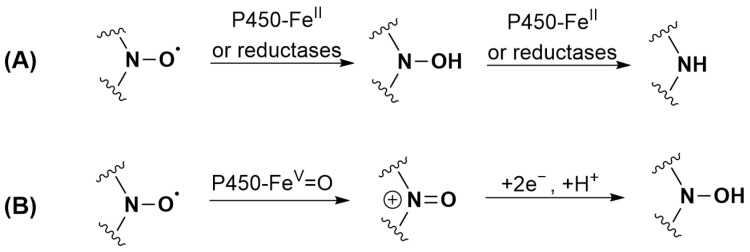
Proposed transformations of nitroxides: (**A**) **1** (TEMPONE) and **5** by P450 under anaerobic conditions; (**B**) **1** (TEMPONE) and **2** when incubated with RLM and NADPH under aerobic conditions.

P450 is mainly known for its monooxygenase activity. However, it can also perform reduction reactions in the absence [53,74] as well as in the presence of oxygen [75]. The substrate that reaches the P450 heme active site can be reduced by heme-Fe^II^, as the standard reduction potential at pH 7.0 (E^0′^) of the ferric/ferrous couple is close to −300 mV/NHE [76,77]. The reduction can be inhibited by Clo. Indeed, the change in the redox potential of heme iron associated with the binding of the strong sixth ligand Clo is known to make the formation of heme-Fe^II^ by the reductase more difficult [78]. A direct reaction of the flavoprotein reductases cannot be excluded for TEMPONE **1** as the reaction is only partially inhibited by adding Clo. As shown in the literature, nitroxide reduction does not end with the formation of hydroxylamines but can also irreversibly lead to the formation of amines, although the exact reaction mechanism is unknown [79]. Both products were identified in the case of nitroxide **5**. The proposed transformations of nitroxides **1** (TEMPONE) and **5** by P450 under anaerobic conditions are shown in Figure 4A.

The fact that tetraethyl-substituted nitroxides appeared less stable than tetramethyl nitroxide **2** in the present situation contradicts the assumption that tetraethyl substitution affords protection against reduction in a biological context. Knowing that lipophilicity is extremely important to the P450 substrate binding affinity and relative catalytic rate [80], this is not surprising as nitroxide **2** appeared rather hydrophilic with a negative $logD_{7.4}$ value (Figure 1) and was expected not to bind significantly to P450.

### 3.3. Aerobic Study of Nitroxides in Incubation with RLM

The kinetics of the same five nitroxides (Figure 1) when incubated with RLM enriched in NADPH, were further investigated in the presence of oxygen and recorded by EPR for 30 min. The initial decrease rates are reported in Table 3.

As shown in Table 3, the initial decay rates of all nitroxides except **2** were lower when oxygen was present in the system. This is in partial accordance with previous reports that the reduction of nitroxides in microsomes and cells is strongly slowed down by the presence of oxygen [40]. For all structures under study, the reaction was dependent on both microsomes and NADPH. DPI and Clo totally inhibited the reaction of tetraethyl nitroxides **3**, **4** and **5**, while only partial inhibition was observed with tetramethyl nitroxides **1** and **2**.

By contrast with what was observed with TEEPONE [41], tetraethyl-substituted nitroxides showed increased stability compared to tetramethyl-substituted compounds. TEMPONE **1** (15%) and nitroxide **2** (42%) were partially recovered upon reoxidation with potassium ferricyanide suggesting partial conversion to hydroxylamine, whereas this was not the case for nitroxides **3**, **4** and **5**. Reduction is thus not a significant route of transformation for tetraethyl nitroxides under these conditions. For tetramethyl-substituted nitroxides **1** and **2**, the reduction mechanism involving heme-Fe^II^ previously proposed in Figure 4A is unlikely under aerobic conditions due to the high affinity of heme-Fe^II^ for oxygen. A direct reduction by reductases or a sequential mechanism corresponding to an initial oxidation to the oxoammonium cation by heme-Fe^V^ = O (1.2 < E^0^ < 2.0 V/NHE [79]) followed by a two-electron reduction by NADPH could occur (Figure 4B) [19,79]. The ability of nitroxides to be readily oxidized to the oxoammonium cation by hypervalent heme complexes that are formed via reaction of heme proteins with H_2_O_2_ is indeed evidenced in the literature [81,82,83,84] but has never been proposed in the reaction with P450 to the best of our knowledge. We did not investigate the reactions of nitroxides **1** and **2** further as studies are already present in the literature [40,85].

**Figure 5:**
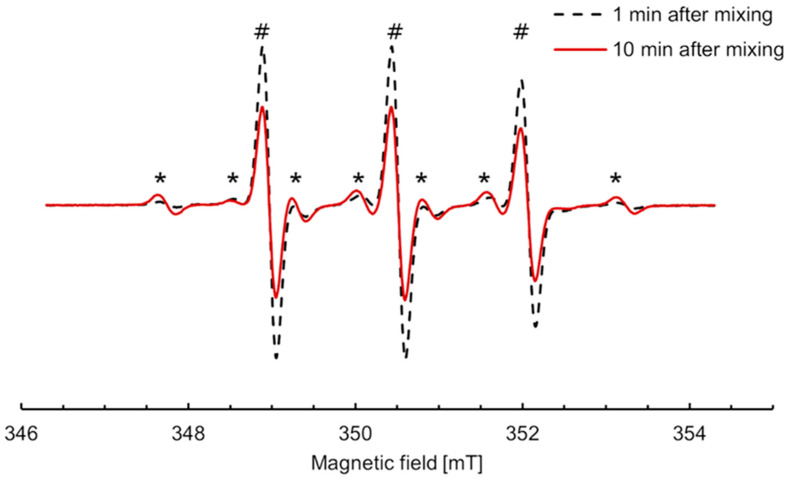
EPR spectra of nitroxide **5** (300 µM) incubated with RLM (30 µM P450) supplemented with NADPH (2 mM) in potassium phosphate buffer (0.1 M, pH 7.4, containing 1 mM DTPA) in the presence of oxygen at 21 °C, 1 min (black dash) and 10 min (red line) after mixing, respectively. The intensity of the new six-line species (*) increased concomitantly to the decrease in the three lines characteristic of nitroxide **5** (#).

The shape of the EPR spectra of incubation mixtures containing nitroxide **5** changed by opposition to all other studied nitroxides. Conditions were optimized so that the conversion to the new paramagnetic species increased and afforded easier characterization. The EPR spectra upon incubation of 300 μM nitroxide **5** with RLM (30 μM P450) and 2 mM NADPH under aerobic conditions at 21 °C are shown in Figure 5. The spectral properties were simulated using EasySpin in Matlab (Figure S6), and data extracted from the simulation are shown in Supplementary Table S2. The three lines characteristic of nitroxide **5** in an aqueous environment (A_N_ = 1.55 mT) were superimposed with six new lines, indicating that the single electron coupled with a hydrogen atom in addition to the nitrogen in the new species (A_N_ = 1.58 mT, A_H_ = 2.36 mT). The intensity of the six new lines increased up to 10 min, corresponding to 7% of the initial nitroxide, then began to decrease, suggesting that the new species was an intermediate in the transformation.

**Figure 6:**
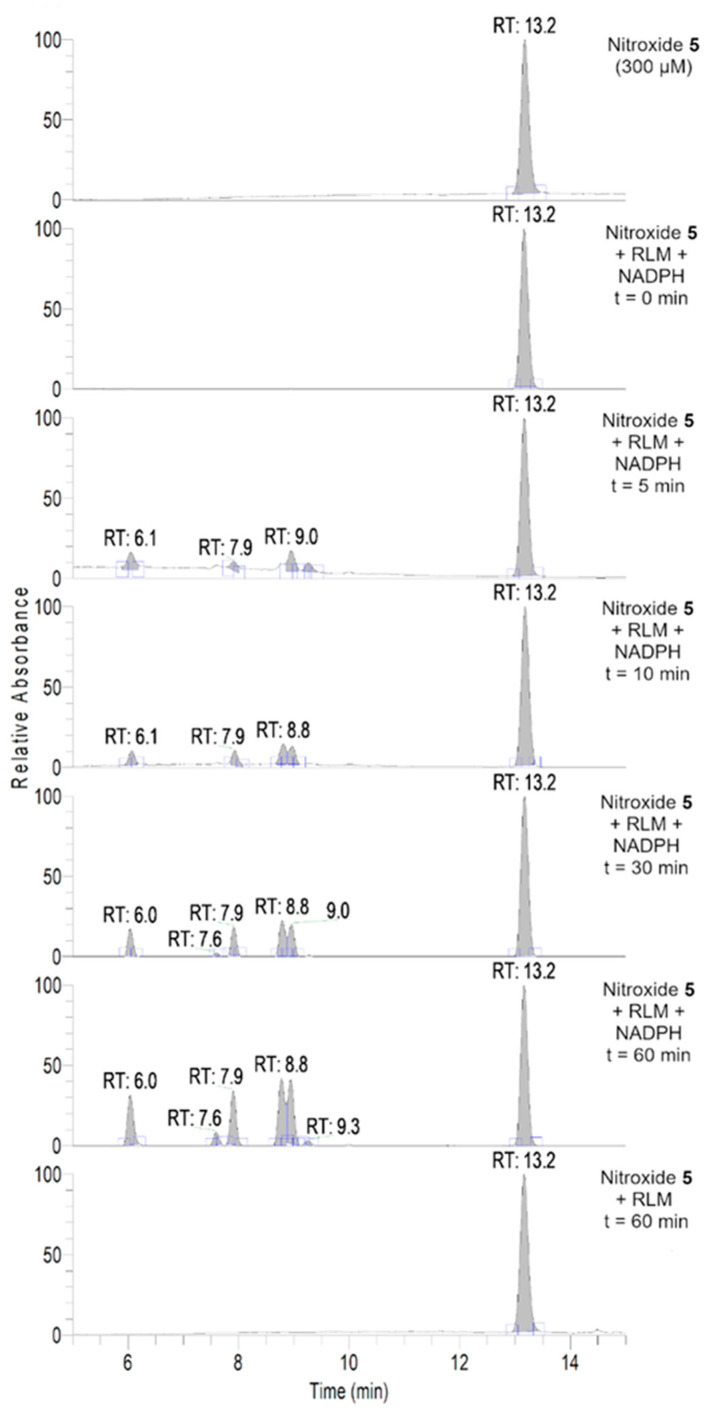
Total scan UV-Vis chromatograms of incubations of nitroxide **5** (300 µM) with RLM (30 µM of P450) with and without NADPH (2 mM) in potassium phosphate buffer (0.1 M, pH 7.4) in the presence of oxygen at 21 °C at different time points. Samples were prepared and analyzed as described in the experimental section.

To gain a better insight into the reaction pathway of nitroxide **5** with RLM in the presence of oxygen, a HPLC-HRMS study was performed as explained in the experimental section. The obtained total scan UV-Vis chromatograms and reconstructed chromatograms of the major ions and their mass spectra are shown in Figure 6 and Figure S7. The main absorption bands of the products are displayed in Table S3 in the Supplementary Material.

**Figure 7:**
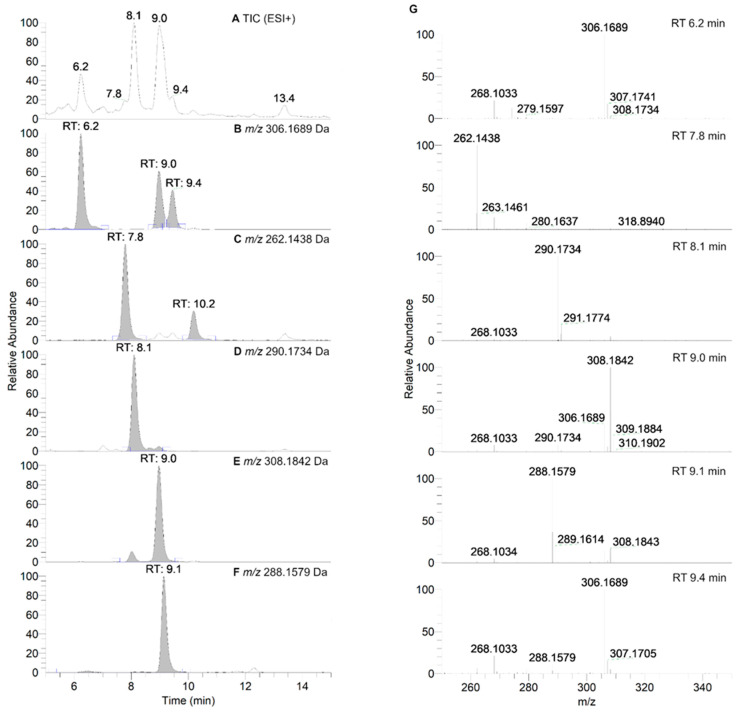
HPLC-HRMS study of nitroxide **5** (300 µM) incubated with RLM (7.8 mg·mL^−1^ protein, 30 µM P450) supplemented with NADPH (2 mM) in potassium phosphate buffer (100 mM; pH 7.4) upon aerobic conditions after 60 min. (**Panel A**) shows the TIC chromatogram in ESI^+^. (**Panels B**–**F**) display reconstructed chromatograms of the principal ions in ESI^+^ mode identified at *m*/*z* = 306.1689, 262.1438, 290.1734, 308.1842, and 288.1579 Da, respectively. (**Panel G**) represents the HRMS spectra of the peaks at retention times (RT) = 6.2, 7.8, 8.1, 9.0, 9.1 and 9.4 min. Peaks in TIC chromatograms were observed with a delay of 0.2 min compared to the UV-Vis detection.

As can be seen in Figure 6, nitroxide **5** eluted at about 13.2 min and was accompanied by six more peaks after 60 min of incubation (summarized in Table 4). The compound eluting at 9.3 min appeared 5 min after the start of the reaction and decreased over time, indicating that the corresponding compound was an intermediate, while other products appeared more stable. The absence of hydroxylamine product at RT = 10.9 min was confirmed. Nitroxide **5** absorbed at 240 nm, and after analyzing the UV spectra of the detected compounds, we can conclude that some of the products were characterized by a more extended delocalization in their structure, as the absorption of the compounds eluting at 7.6, 7.9, and 9.0 min were red-shifted with bands at 312, 273 and 272 nm, respectively (Supplementary Material, Table S3). All the products were more easily ionized in ESI^+^ (Figure 7) than in ESI^−^ mode (Supplementary Material, Figure S7), and thus behaved differently from the starting material.

**Figure 8:**
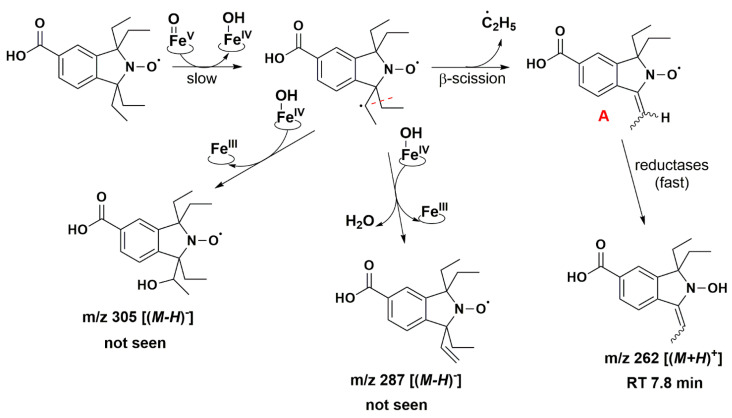
Proposed reaction pathways in the aerobic transformation of nitroxide **5** by RLM and NADPH, part I. Explicit hydrogen in structure **A** corresponds to potential origin of the coupling seen in the EPR spectra. Initial hydrogen abstraction could occur on either of the ethyl groups leading to isomers. Only one possibility is presented for the sake of clarity. The given RT values correspond to the reconstructed chromatograms of the principal ions obtained in HRMS study and are characterized by a delay of 0.2 min compared to the UV-Vis detection (see Figure 7).

Isoindoline-based nitroxide with tetraethyl substitution was converted into several compounds, one of which was paramagnetic and unstable, as shown by the EPR and HPLC-HRMS data. Based on P450 known oxidase activity [79,86] and previous results with TEEPONE [41], we propose that P450-Fe^V^ = O activated the substrate (nitroxide **5**) by abstracting a hydrogen atom from one of the ethyl groups (E^0′^ ≈ 1.22 V/ENH for the P450 Fe^V^ = O/Fe^IV^ - OH couple at pH 7) [87]. This step is slow. Once it occurs, usual P450 reactions would lead to hydroxylation through the “oxygen rebound” mechanism [88] or desaturation as a competitive process [89] (formally, a second dehydrogenation by abstraction of an electron plus a proton by P450 - Fe^IV^ - OH). The corresponding nitroxide products proposed in Figure 8 do not seem to have been formed in significant proportions since we would expect them to be easily detected in negative mode.

**Figure 9:**
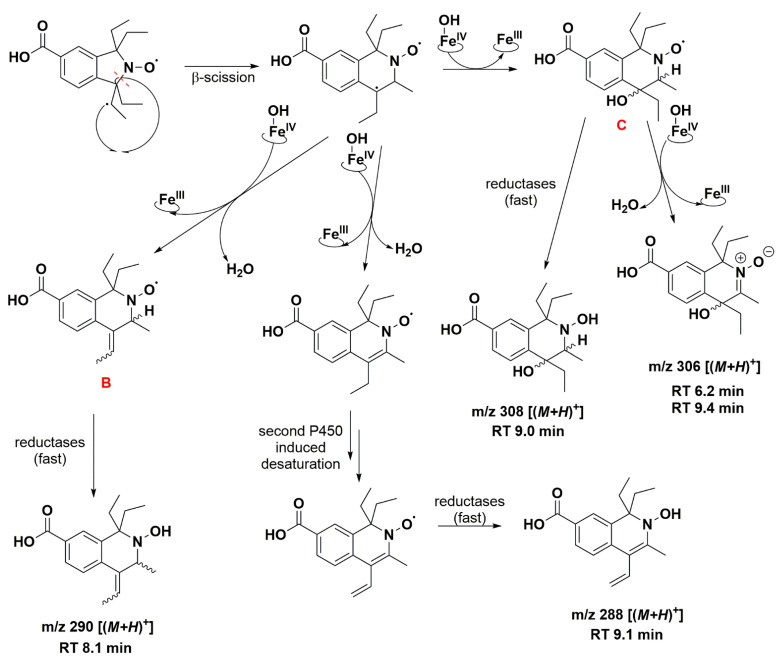
Proposed reaction pathway in the aerobic transformation of nitroxide **5** by RLM and NADPH, part II. Explicit hydrogens attached to carbon atoms in structures **B** and **C** correspond to potential origin of the coupling seen in the EPR spectra. Only one possible isomer is presented at each stage for the sake of clarity. The given RT values correspond to the reconstructed chromatograms of the principal ions obtained in HRMS study and were characterized by a delay of 0.2 min compared to the UV-Vis detection (see Figure 7).

**Figure 10:**
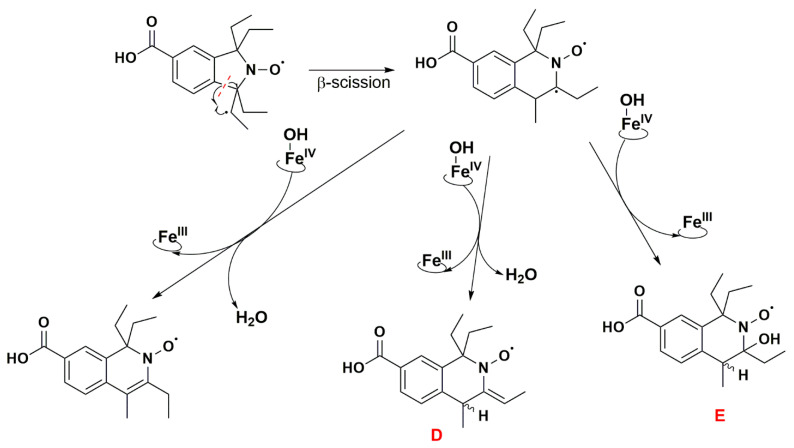
Proposed reaction pathway in the aerobic transformation of nitroxide **5** by RLM and NADPH, part III. Explicit hydrogens attached to carbon atoms in structures **D** and **E** correspond to potential origin of the coupling seen in the EPR spectra. The given RT values correspond to the reconstructed chromatograms of the principal ions obtained in HRMS study and were characterized by a delay of 0.2 min compared to the UV-Vis detection (see Figure 7).

The absence of such products suggests that once the first dehydrogenation on one ethyl group has occurred, the resulting radical could undergo rapid β-scission reactions, which would contribute to reducing the steric strain in the molecule, followed by further reactions characteristic of P450 [79]. One possibility of β-scission corresponds to the loss of an ethyl radical, yielding nitroxide **A** presented in Figure 8. The latter was no longer sterically shielded and the product detected at RT 7.6 min was compatible with the hydroxylamine form (*m*/*z* 262 Da for [(*M+H*)*^+^*]) obtained after enzymatic reduction in the microsomal incubation. The absorption band at 312 nm further supports this hypothesis (Supplementary Table S2). Two other β-scission reactions leading to a ring expansion can be proposed as shown in Figure 9 and Figure 10. The pathway in Figure 9 is more probable, thanks to resonance-induced stabilization of the tertiary carbon radical in benzylic position. Further desaturation by P450-Fe^IV^-OH (E^0′^ ≈ 0.99 V/NHE) and enzymatic reduction could explain the products detected at RT = 7.9 and 8.8 min with absorption bands around 270 nm, and *m*/*z* 290 and 288 Da for [(*M+H*)*^+^*], respectively. Hydroxylation by P450-Fe^IV^-OH could yield structure **C**, which could either be reduced to the corresponding hydroxylamine (consistent with the peak at RT = 9.0 min and *m*/*z* 308 Da for [(*M+H*)*^+^*]), or be oxidized to the nitrone (*m*/*z* 306 Da for [(*M+H*)*^+^*]). Peaks at RT = 6.0 and 9.2 min could correspond to different isomers.

Structures **A**, **B**, **C**, **D** and **E** (Figure 8, Figure 9 and Figure 10) are potential candidates for the new paramagnetic species, which is transient and contains a hydrogen atom in close vicinity of the unpaired electron, leading to an extra splitting in the EPR spectra. DFT calculations were performed to confirm which β-scission reaction pathway was the more favorable and to identify the structure of the paramagnetic intermediate. First and foremost, the structure of nitroxide **5** was subjected to geometry optimization using the B3LYP/G functional and used to benchmark the DFT results with respect to the nitrogen hfcc. Solvent effects were induced using the CPCMC approach with water as a solvent. Different basis sets were tested and the 6–31 g* provided the fairest agreement between experiment and theory (Supplementary Table S4). However, as the computed nitrogen hfcc (37.2 MHz/1.33 mT) was underestimated when compared to the experimental value (43.4 MHz/1.55 mT), we attempted to improve our predictions by considering new DFT models including one, two and three explicit water molecules in the surrounding of the N-O group in nitroxide **5** (Supplementary Figure S8), following a methodology similar to that used in our previous study [41]. We verified that the corresponding structures converged to a real minimum by performing numerical frequency calculations. Calculations were found converged when one single water molecule was considered and the inclusion of additional solvent molecules did not further improve the predictions (Supplementary Table S5). A value of 41.6 MHz/1.49 mT was obtained for the computed hfcc of nitroxide **5** model with one explicit water molecule, in good agreement with the experiment. Furthermore, given that at pH 7.4 nitroxide **5** was deprotonated, we optimized the geometry of nitroxide **5** in carboxylate form with one explicit water molecule in the surroundings of the NO group and consistent EPR parameters were computed (A_N_ = 42.1 MHz/1.50 mT). Based on these results, we pursued our investigations considering the possible compounds **A** to **E** interacting with a single explicit water molecule. For species **A**, **B**, **D** and **E,** we obtained computed hfccs that did not agree with the experimental ones (Supplementary Table S6). Structure **C** may exist as *trans* and *cis* isomers when considering the position of the explicit hydrogen in Figure 9 and the hydroxyl group relative to the saturated ring. The structure was more flexible than nitroxide **5** and the A_H_ value was highly sensitive to the ring conformation. The calculated hfccs for the *trans* isomer of structure **C** were in good agreement with the experimental hfcc of the new paramagnetic species, while those of the *cis* isomer were out of range. Following the same procedure as for nitroxide **5**, structure **C** *trans* in carboxylate form with one explicit water molecule was optimized and its theoretical EPR parameters were determined. From the results summarized in Table 5, we observed that the computed hfccs for both protonated and deprotonated forms of structure **C** *trans* were of similar magnitude which is consistent with the results obtained for nitroxide **5** in both carboxylic and carboxylate forms.

**Figure 11:**
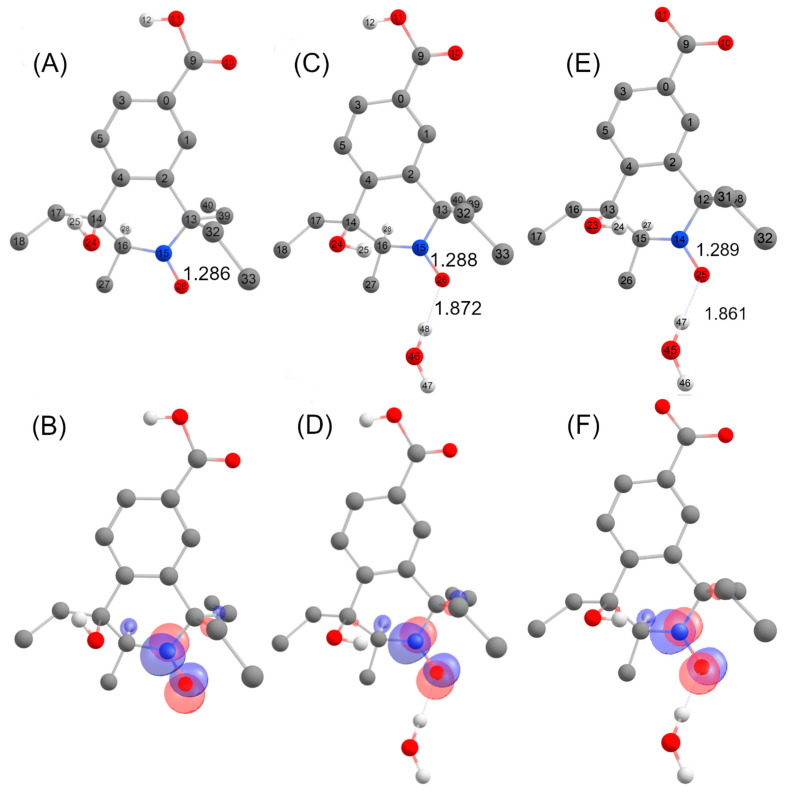
DFT- optimized structures with atom labelling together with selected metrical parameters (N-O and O-H bond distances, Å) for structures **C** (carboxylic acid form) (**A**), **C** (carboxylic acid form) + H_2_O (**C**), **C** (carboxylate form) + H_2_O (**E**) and localized SOMOs of structures **C** (carboxylic acid form) (**B**), **C** (carboxylic acid form) + H_2_O (**D**) and **C** (carboxylate form) + H_2_O (**F**).

The DFT-optimized geometries and the singly occupied molecular orbitals (SOMO) of **C** *trans* with and without one explicit water molecule are shown in Figure 11. The unpaired electron was mainly delocalized over the N-O moiety and occupied an antibonding π-orbital in both cases, while the SOMO also partially extended on the neighboring C-H group accounting for the large A_H_ value in the corresponding EPR spectrum. The identification of structure **C** *trans* as the transient species observed by EPR supports the β-scission reaction pathway displayed in Figure 9 as a probable route of transformation.

The conversions of nitroxides **3** and **4** by P450 were negligible under aerobic conditions, with 5–10% decay in 30 min (Table 3), indicating that these five-membered tetraethyl-substituted nitroxides were the most stable of the compounds studied here. The difference in reactivity observed with compound **5** was likely due to a reduced lipophilicity ($logD_{7.4}$ = 0.362 and −1.054 for **3** and **4**, respectively, versus 1.295 for compound **5**, Figure 1).

## 4. Discussion

The present study offers a deeper insight into the reactions that control the biological stability of tetraethyl-substituted nitroxides. It was performed using a set of nitroxides with different ring structures and substitutions but extrapolation to other compounds should be carried out with caution. Keeping this in mind, it emerges that the rate of reduction by ascorbate, though an important criterion to guide future probe design, is not the only parameter to take into account as it may appear as a minor factor in the transformations of nitroxides in biological systems [90]. Except in specific cell types such as hepatocytes, neurons and renal cells, where the concentration of ascorbate can reach 10 mM or higher, the ascorbate level is rather low within most tissues (1–5 mM) or in plasma (50 µM) [40,91].

The metabolism of nitroxides in microsomes is complex, resulting in various products and is highly dependent on the lipophilicity of the probe. Previously, it was reported that the transformation of nitroxides occurs faster under anaerobic conditions than under aerobic conditions when incubated with RLM and NADPH [40]. Nitroxide structures examined in this study followed this trend, with the exception of structure **2**. This is important because normal tissue oxygen levels vary within and among organs in the range of 3–9%, fairly lower than the 21% present in air or in standard cell cultures. Moreover, rapidly proliferating tumors are known to be characterized by hypoxic regions.

**Figure 12:**
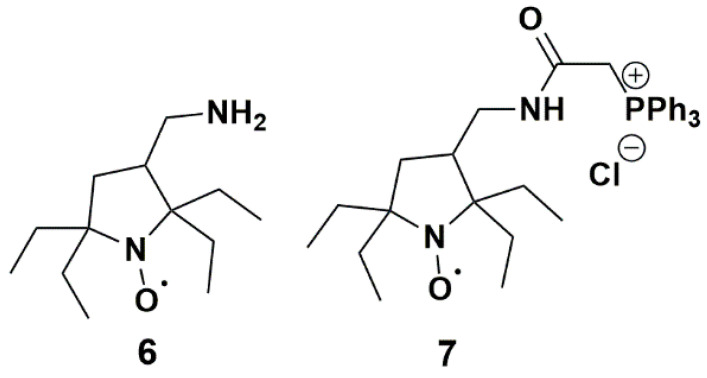
Pyrrolidine nitroxides tested by Kirilyuk et al.; (**6**) 3-(aminomethyl)-2,2,5,5-tetraethylpyrrolidineyloxyl; (**7**) 3-(2-triphenylphosphonio)acetamidomethyl-2,2,5,5-tetraethylpyrrolidine-1-oxyl chloride.

Nitroxides **3** and **4** showed the most significant resistance to reduction by ascorbate and conversion by P450, probably due to steric shielding and poor affinity for P450. Therefore, they could appear as ideal candidates for in vivo applications. Yet, nitroxide **3** had a large linewidth (0.38 mT), which impaired the resolution of EPR imaging [92], while synthetic yields of nitroxide **4** in the described procedures were low [48] and poorly compatible with quantities currently required for in vivo EPR. Moreover, nitroxides **3** and **4** are unlikely to be used as such in vivo as their negative charge would prevent penetration within cells. We can expect that derivatives of nitroxide **3** and **4** with higher lipophilicity would demonstrate a faster reaction with P450 present in the liver and different organs. Interestingly, Kirilyuk et al. [29] recently prepared reduction-resistant spin labels and probes in the pyrrolidine series and studied the decay of compounds **6** and **7** (Figure 12) in homogenates of liver and other organs. Compound **6** had a basic primary amine substituent which mainly existed in the protonated form at physiological pH. This nitroxide had comparable lipophilicity with **3** and **4**, while compound **7** was more lipophilic and able to permeate cellular membranes thanks to the lipophilic triphenylphosphonium cationic group. Both nitroxides significantly decayed in liver homogenates but the kinetics were more pronounced for nitroxide **7**, which could be related to a higher affinity for P450.

Because it was transformed at a slightly higher rate than other tetraethyl nitroxides, it was possible to obtain further insights into the mechanism of transformation of nitroxide **5** by P450 under different conditions. It is important to keep in mind that the conversion of nitroxide **5** was amplified in our study because the concentration of P450 in the system was exaggerated in comparison with physiological conditions. This isoindoline-based tetraethyl-substituted nitroxide retained increased stability overall compared to tetramethyl-substituted nitroxides with six- and five-membered rings. By combining EPR and HPLC-HRMS data, we demonstrated that nitroxide **5** was slowly reduced to hydroxylamine and amine under anaerobic conditions, with the involvement of P450-Fe^II^ being most likely. In contrast to hydroxylamine, the amine cannot be reoxidized to the nitroxide using ferricyanide. Oxidation of sterically hindered secondary amines by monooxygenase reaction in the liver has, however, been observed with tetramethyl-substituted compounds [93], and it would be interesting to investigate the possibility of a similar hepatic redox cycling in the present case.

The metabolism of nitroxide **5** was completely different when oxygen was present. The crucial step was hydrogen abstraction by the P450-Fe^V^ = O complex. According to the literature and our observations, this step was slow, and it was DFT-calculated that hydrogen abstraction from secondary carbon atoms was more favorable than from primary ones [94]. For this reason, we assumed that hydrogen abstraction occurred at one of the ethyl groups on the secondary carbon atom. It was followed by β-scissions rather than hydroxylation or desaturation reactions, leading to significative modifications of the core of the probe. The presence of the aromatic ring likely drove these reactions through the stabilization of an intermediate radical at benzylic position and by enhancing the lipophilic character of the probe and its affinity for P450 active site.

The fact that nitroxide **5** was metabolized and converted into several different compounds at a slow rate would suggest that detoxification routes exist and that such a probe (or its derivatives) would not persist in the organism over undesired periods of time, which could prevent any long-term detrimental effects and be desirable in some in vivo applications such as EPR imaging.

## 5. Conclusions

Since more and more tetraethyl-substituted nitroxides are being designed for biological and in vivo studies, it was of interest to investigate their metabolic stability upon reaction with P450 in a comparative study involving different cores and substitutions (Figure 1) with the purpose of identifying stabilizing structural elements. We confirmed that despite the protection afforded against ascorbate-induced reduction, the inclusion of a tetraethyl-substituted scaffold in the probe candidate is not a guarantee for high stability in vivo. A negative charge and/or minimal hydrophilicity is required to slow down transformation by P450 enzymes, either via reductive or oxidative metabolism.

## Figures and Tables

**Table 1:** Initial second-order reaction rate constants (M^−1^·s^−1^) for the reduction of a series of nitroxides (Figure 1) by excess ascorbate.

Nitroxide	k_0_ (M^−1^·s^−1^)	Reference
**1**	6.9 ± 0.1	This work
	6.9 ± 0.7	[57]
	5.75 ± 0.04	[42]
	7.2 ± 0.8 ^a^	[70]
**2**	0.072 ± 0.003	This work
	0.11 ± 0.01	[70]
	0.079 ± 0.001	[42]
	0.060 ± 0.003 ^a^	[71]
**3**	0.0011 ± 0.0002	This work
	≤0.001	[30]
	0.002 ± 0.001	[42]
	0.0011 ± 0.0002 ^a^	[71]
**4**	0.0077 ± 0.0009	This work
	0.00122 ± 0.00003 ^a^	[71]
**5**	0.02 ± 0.01	This work

^a^ Reaction measured with ascorbate and glutathione mixtures.

**Table 2:** Initial rates of decrease (nmol·min^−1^·mg^−1^·prot) ((nmol·min^−1^·nmol^−1^·P450)) of examined nitroxides (100 μM) in the presence of rat liver microsomes (RLM), and various cofactors and inhibitors (Clo: clotrimazole; DPI: diphenyliodonium chloride) upon anaerobic conditions (*** = *p* < 0.001, ** *p* < 0.01 and * = *p* < 0.05 for difference from whole system (WS)). The samples were prepared in potassium phosphate buffer (100 mM, pH 7.4, containing 1 mM DTPA). WS corresponds to the nitroxide incubated with RLM and NADPH added the last to initiate the reaction. The incubations were prepared at 21 °C. The samples were flushed with nitrogen until oxygen was removed completely.

Nitroxide	Anaerobic				
	WS	-RLM	-NADPH	+DPI	+ Clo
TEMPONE **1**	19.3 ± 0.9 (5.0 ± 0.5)	0.0 **	0.0 **	2.7 ± 0.3 ** (0.70 ± 0.08)	4.3 ± 0.4 (1.1 ± 0.2)
**2**	0.0	0.0	0.0	0.0	0.0
**3**	2.2 ± 0.6 (0.56 ± 0.08)	0.0	0.0	0.0	0.0
**4**	1.8 ± 0.2 (0.47 ± 0.05)	0.0 *	0.0 *	0.0 *	0.0 *
**5**	5.2 ± 0.5 (1.35 ± 0.07)	0.0 ***	0.0 ***	0.0 ***	0.0 ***

**Table 3:** Initial rates of decrease (nmol·min^−1^·mg^−1^ prot) ((nmol·min^−1^·nmol^−1^ P450)) of examined nitroxides (100 µM) in the presence of RLM and various cofactors and inhibitors upon aerobic conditions (*** = *p* < 0.001, ** *p* < 0.01 and * = *p* < 0.05 for difference from aerobic WS, except for aerobic WS for which difference from anaerobic WS in Table 2 is given). The samples were prepared in potassium phosphate buffer (100 mM, pH 7.4, containing 1 mM DTPA). WS corresponds to the nitroxide incubated with RLM and NADPH added the last to initiate the reaction. The incubations were prepared at 21 °C.

Nitroxide	Aerobic				
	WS	-RLM	-NADPH	+DPI	+ Clo
TEMPONE **1**	11.1 ± 0.8 * (2.8 ± 0.2)	0.0 ***	0.0 ***	5.1 ± 0.3 ** (1.31 ± 0.07)	1.2 ± 0.3 *** (0.30 ± 0.09)
**2**	4.8 ± 0.8 ** (1.2 ± 0.1)	0.0 **	0.0 **	2.1 ± 0.3 * (0.54 ± 0.07)	0.94 ± 0.09 * (0.24 ± 0.03)
**3**	1.8 ± 0.2 (0.46 ± 0.05)	0.0 **	0.0 **	0.0 **	0.0 **
**4**	0.84 ± 0.09 ** (0.22 ± 0.03)	0.0 **	0.0 **	0.0 **	0.0 **
**5**	3.4 ± 0.2 * (0.88 ± 0.05)	0.0 ***	0.0 ***	0.0 ***	0.0 ***

**Table 4:** HPLC-HRMS characteristics of the main products observed in the incubation of nitroxide **5** (300 µM) with RLM (7.8 mg·mL^−1^ protein, 30 µM P450) supplemented with NADPH (2 mM) upon aerobic conditions. The given RT values correspond to the reconstructed chromatograms of the principal ions obtained in HRMS study and were characterized by a delay of 0.2 min compared to the UV-Vis detection (see Figure 7).

RT [min]	Positive ESI Mode			Suggested Modification
	*m*/*z* [Da]		Chemical Formula	
	Observed	Calculated		
6.2	306.1689	306.1700	C_17_H_24_NO_4_, [(*M+H*)^+^]	+O, −H
7.8	262.1438	262.1438	C_15_H_20_NO_3_, [(*M+H*)^+^]	−C_2_H_6_, +H
8.1	290.1734	290.1751	C_17_H_24_NO_3_, [(*M+H*)^+^]	−H_2_, +H
9.0	308.1842	308.1856	C_17_H_26_NO_4_, [(*M+H*)^+^]	+O, +H
9.1	288.1579	288.1594	C_17_H_22_NO_3_, [(*M+H*)^+^]	−2H_2_, +H
9.4	306.1689	306.1700	C_17_H_24_NO_4_, [(*M+H*)^+^]	+O, −H

**Table 5:** DFT-calculated hyperfine coupling constants (hfccs, MHz/mT) and comparison to experimental results. The hydrogen atom under study is explicitly presented in Figure 9.

Compound	A_N_ (MHz/mT)	A_H_ (MHz/mT)
5 (carboxylic acid form)	37.2/1.33	-
5 (carboxylic acid form) + H_2_O	41.6/1.49	-
5 (carboxylate form) + H_2_O	42.1/1.50	-
5 (Expt.)	43.4/1.55	-
C *trans* (carboxylic acid form)	41.3/1.47	70.3/2.51
C *trans* (carboxylic acid form) + H_2_O	42.5/1.52	73.4/2.62
C *trans* (carboxylate form) + H_2_O	42.9/1.53	73.9/2.64
6-line intermediate (Expt.)	44.3/1.58	66.1/2.36

## Data Availability

The data presented in this study are available upon request from the corresponding author.

## Supplementary Materials

The following supporting information can be downloaded at: https://www.mdpi.com/article/10.3390/antiox12020402/s1, Figure S1: Total scan UV-Vis, TIC (ESI^−^) and (ESI^+^) chromatograms of nitroxide **5**; Figure S2: HRMS spectrum of nitroxide **5** in positive and negative mode; Figure S3: Superhyperfine structure of nitroxide **5** in potassium phosphate buffer in the absence of oxygen; Figure S4: Total scan UV-Vis, TIC (ESI^−^) and (ESI^+^) chromatograms of the incubations of nitroxide **5** with sodium L-ascorbate in pure water upon anaerobic conditions; Figure S5: HRMS spectra of the hydroxylamine product of the anaerobic incubation of nitroxide **5** with sodium L-ascorbate in pure water obtained in ESI^−^ and ESI^+^ modes, respectively; Figure S6: Experimental spectrum of nitroxide **5** incubated with RLM enriched in NADPH after 10 min under aerobic conditions overlapped with the simulated EPR spectrum; Figure S7: HPLC-HRMS study of nitroxide **5** incubated with RLM supplemented with NADPH in potassium phosphate buffer upon aerobic conditions after 60 min; Figure S8: DFT-optimized structures for nitroxide **5** without, with one, two, and three explicit water molecules and in carboxylate form with one explicit water molecule together with selected metrical parameters and localized SOMOs; Table S1: ClogP, pKa and $logD_{7.4}$ values calculated for ionizable compounds under study; Table S2: EPR spectral characteristics of identified species observed in the reaction of nitroxide **5** with RLM and NADPH; Table S3: Main absorption bands in the UV spectra from HPLC-HRMS study of nitroxide **5** incubated with RLM enriched in NADPH in potassium phosphate buffer under aerobic conditions; Table S4: DFT-computed hyperfine coupling constants of nitroxide **5** using the B3LYP/G functional and various basis sets; Tables S5 and S6: Free energies and DFT-computed nitrogen hyperfine coupling constants of the nitroxide structures under study; Tables S7 to S24: DFT-optimized Cartesian coordinates for the nitroxide structures under study.

## Abbreviations

A_H_, isotropic hydrogen hyperfine coupling constant; A_N_, isotropic nitrogen hyperfine coupling constant; BSA, bovine serum albumin; B3LYP/G, Becke 3-parameter Lee-Yang-Parr; Clo, clotrimazole; CPCMC, conductor-like polarizable continuum model; ClogP, calculated n-octanol/water partition coefficient; DFT, density functional theory; DMSO, dimethyl sulfoxide; DPI, diphenyliodonium chloride; DTPA, diethylenetriaminepentaacetic acid; E^0^, standard reduction potential relative to normal hydrogen electrode (NHE); E^0′^, standard reduction potential relative to NHE at pH 7.0; EPR, electron paramagnetic resonance (equivalent to ESR, electron spin resonance); ESI^+^, positive electrospray ionization; ESI^−^, negative electrospray ionization; FAD, flavin adenine dinucleotide; FMN, flavin mononucleotide; hfcc, hyperfine coupling constant; HPLC, high performance liquid chromatography; HRMS, high resolution mass spectrometry; $logD_{7.4}$, distribution coefficient at pH 7.4; MRI, magnetic resonance imaging; NADH, reduced nicotinamide adenine dinucleotide; NADPH, reduced nicotinamide adenine dinucleotide phosphate; P450, cytochrome P450; RLM, rat liver microsomes; rpm, rotations per minute; RT, retention time; SCF, self-consistent field; SOD, superoxide dismutase; SOMO, singly occupied molecular orbital; TEEPOL, 2,2,6,6-tetraethyl-4-hydroxy(piperidine-1-yloxyl; TEEPONE, 2,2,6,6-tetraethyl-4-oxo-piperidinyloxyl; TEMPONE, 2,2,6,6-tetramethyl-4-oxo-piperidineyloxyl; TIC, total ion current; UV-Vis, ultraviolet-visible; WS, whole system.

## References

[1] Samuni Y., Gamson J., Samuni A., Yamada K., Russo A., Krishna M.C., Mitchell J.B. (2004). Factors Influencing Nitroxide Reduction and Cytotoxicity In Vitro. Antioxid. Redox Signal..

[2] Hosokawa K., Chen P., Lavin F.M., Bottle E.S. (2004). The Impact of Carboxy Nitroxide Antioxidants on Irradiated Ataxia Telangiectasia Cells. Free Radic. Biol. Med..

[3] Kocherginsky N., Swartz H.M. (1995). Nitroxide Spin Labels: Reactions in Biology and Chemistry.

[4] Zamora P.L., Villamena F.A., Berliner L.J., Parinandi N.L. (2020). Clinical Probes for ROS and Oxidative Stress. Measuring Oxidants and Oxidative Stress in Biological Systems.

[5] Fujii H., Emoto M., Sato-Akaba H. (2019). Brain Redox Imaging Using In Vivo Electron Paramagnetic Resonance Imaging and Nitroxide Imaging Probes. Magnetochemistry.

[6] Ilangovan G., Li H., Zweier J.L., Kuppusamy P., Vallyathan V., Shi X., Castranova V. (2002). In Vivo Measurement of Tumor Redox Environment Using EPR Spectroscopy. Oxygen/Nitrogen Radicals: Cell Injury and Disease.

[7] Elas M., Ichikawa K., Halpern H.J. (2012). Oxidative Stress Imaging in Live Animals with Techniques Based on Electron Paramagnetic Resonance. Radiat. Res..

[8] Bačić G., Pavićević A., Peyrot F. (2016). In Vivo Evaluation of Different Alterations of Redox Status by Studying Pharmacokinetics of Nitroxides Using Magnetic Resonance Techniques. Redox Biol..

[9] Babić N., Peyrot F. (2019). Molecular Probes for Evaluation of Oxidative Stress by In Vivo EPR Spectroscopy and Imaging: State-of-the-Art and Limitations. Magnetochemistry.

[10] Ahmad R., Kuppusamy P. (2010). Theory, Instrumentation, and Applications of Electron Paramagnetic Resonance Oximetry. Chem. Rev..

[11] Kirilyuk I.A., Bobko A.A., Khramtsov V.V., Grigor’ev I.A. (2005). Nitroxides with Two PK Values—Useful Spin Probes for pH Monitoring within a Broad Range. Org. Biomol. Chem..

[12] Kovaleva E.G., Molochnikov L.S., Golovkina E.L., Hartmann M., Kirilyuk I.A., Grigor’ev I.A. (2013). Dynamics of pH-Sensitive Nitroxide Radicals in Water Adsorbed in Ordered Mesoporous Molecular Sieves by EPR Spectroscopy. Microporous Mesoporous Mater..

[13] Torricella F., Pierro A., Mileo E., Belle V., Bonucci A. (2021). Nitroxide Spin Labels and EPR Spectroscopy: A Powerful Association for Protein Dynamics Studies. Biochim. Biophys. Acta BBA Proteins Proteom..

[14] Matsumoto K., Nakanishi I., Zhelev Z., Bakalova R., Aoki I. (2022). Nitroxyl Radical as a Theranostic Contrast Agent in Magnetic Resonance Redox Imaging. Antioxid. Redox Signal..

[15] Matsumoto K., Hyodo F., Matsumoto A., Koretsky A.P., Sowers A.L., Mitchell J.B., Krishna M.C. (2006). High-Resolution Mapping of Tumor Redox Status by Magnetic Resonance Imaging Using Nitroxides as Redox-Sensitive Contrast Agents. Clin. Cancer Res..

[16] Hyodo F., Chuang K.-H., Goloshevsky A.G., Sulima A., Griffiths G.L., Mitchell J.B., Koretsky A.P., Krishna M.C. (2008). Brain Redox Imaging Using Blood—Brain Barrier-Permeable Nitroxide MRI Contrast Agent. J. Cereb. Blood Flow Metab..

[17] Hodgson J.L., Namazian M., Bottle S.E., Coote M.L. (2007). One-Electron Oxidation and Reduction Potentials of Nitroxide Antioxidants: A Theoretical Study. J. Phys. Chem. A.

[18] Krishna M.C., Russo A., Mitchell J.B., Goldstein S., Dafni H., Samuni A. (1996). Do Nitroxide Antioxidants Act as Scavengers of O2.− or as SOD Mimics?. J. Biol. Chem..

[19] Krishna M.C., Grahame D.A., Samuni A., Mitchell J.B., Russo A. (1992). Oxoammonium Cation Intermediate in the Nitroxide-Catalyzed Dismutation of Superoxide. Proc. Natl. Acad. Sci. USA.

[20] Yamada K.-I., Kuppusamy P., English S., Yoo J., Irie A., Subramanian S., Mitchell J.B., Krishna M.C. (2002). Feasibility and Assessment of Non-Invasive in Vivo Redox Status Using Electron Paramagnetic Resonance Imaging. Acta Radiol..

[21] Emoto M.C., Sato-Akaba H., Hirata H., Fujii H.G. (2014). Dynamic Changes in the Distribution and Time Course of Blood–Brain Barrier-Permeative Nitroxides in the Mouse Head with EPR Imaging: Visualization of Blood Flow in a Mouse Model of Ischemia. Free Radic. Biol. Med..

[22] Bobko A.A., Kirilyuk I.A., Grigor’ev I.A., Zweier J.L., Khramtsov V.V. (2007). Reversible Reduction of Nitroxides to Hydroxylamines: Roles for Ascorbate and Glutathione. Free Radic. Biol. Med..

[23] Emoto M., Mito F., Yamasaki T., Yamada K.-I., Sato-Akaba H., Hirata H., Fujii H. (2011). A Novel Ascorbic Acid-Resistant Nitroxide in Fat Emulsion Is an Efficient Brain Imaging Probe for in Vivo EPR Imaging of Mouse. Free Radic. Res..

[24] Swartz H.M., Sentjurc M., Morse P.D. (1986). Cellular Metabolism of Water-Soluble Nitroxides: Effect on Rate of Reduction of Cell/Nitroxide Ratio, Oxygen Concentrations and Permeability of Nitroxides. Biochim. Biophys. Acta BB Mol. Cell Res..

[25] Swartz H.M., Chen K., Pals M., Sentjurc M., Morse P.D. (1986). Hypoxia-Sensitive NMR Contrast Agents. Magn. Reson. Med..

[26] Azuma R., Yamasaki T., Emoto M.C., Sato-Akaba H., Sano K., Munekane M., Fujii H.G., Mukai T. (2023). Effect of Relative Configuration of TEMPO-Type Nitroxides on Ascorbate Reduction. Free Radic. Biol. Med..

[27] Karthikeyan G., Bonucci A., Casano G., Gerbaud G., Abel S., Thomé V., Kodjabachian L., Magalon A., Guigliarelli B., Belle V. (2018). A Bioresistant Nitroxide Spin Label for In-Cell EPR Spectroscopy: In Vitro and In Oocytes Protein Structural Dynamics Studies. Angew. Chem. Int. Ed..

[28] Braun T.S., Widder P., Osswald U., Groß L., Williams L., Schmidt M., Helmle I., Summerer D., Drescher M. (2020). Isoindoline-Based Nitroxides as Bioresistant Spin Labels for Protein Labeling through Cysteines and Alkyne-Bearing Noncanonical Amino Acids. ChemBioChem.

[29] Dobrynin S.A., Usatov M.S., Zhurko I.F., Morozov D.A., Polienko Y.F., Glazachev Y.I., Parkhomenko D.A., Tyumentsev M.A., Gatilov Y.V., Chernyak E.I. (2021). A Simple Method of Synthesis of 3-Carboxy-2,2,5,5-Tetraethylpyrrolidine-1-Oxyl and Preparation of Reduction-Resistant Spin Labels and Probes of Pyrrolidine Series. Molecules.

[30] Paletta J.T., Pink M., Foley B., Rajca S., Rajca A. (2012). Synthesis and Reduction Kinetics of Sterically Shielded Pyrrolidine Nitroxides. Org. Lett..

[31] Kinoshita Y., Yamada K.-I., Yamasaki T., Sadasue H., Sakai K., Utsumi H. (2009). Development of Novel Nitroxyl Radicals for Controlling Reactivity with Ascorbic Acid. Free Radic. Res..

[32] Kirilyuk I.A., Bobko A.A., Grigor’ev I.A., Khramtsov V.V. (2004). Synthesis of the Tetraethyl Substituted pH-Sensitive Nitroxides of Imidazole Series with Enhanced Stability towards Reduction. Org. Biomol. Chem..

[33] Baranczewski P., Stańczak A., Sundberg K., Svensson R., Wallin A., Jansson J., Garberg P., Postlind H. (2006). Introduction to in Vitro Estimation of Metabolic Stability and Drug Interactions of New Chemical Entities in Drug Discovery and Development. Pharmacol. Rep. PR.

[34] Testa B. (2014). Metabolism in Drug Development. Drug Metabolism Prediction.

[35] Shen J., Bottle S., Khan N., Grinberg O., Reid D., Micallef A., Swartz H. (2002). Development of Isoindoline Nitroxides for EPR Oximetry in Viable Systems. Appl. Magn. Reson..

[36] Quintanilha A.T., Packer L. (1977). Surface Localization of Sites of Reduction of Nitroxide Spin-Labeled Molecules in Mitochondria. Proc. Natl. Acad. Sci. USA.

[37] Chen K., Glockner J.F., Morse P.D.I., Swartz H.M. (1989). Effects of Oxygen on the Metabolism of Nitroxide Spin Labels in Cells. Biochemistry.

[38] Ueda A., Nagase S., Yokoyama H., Tada M., Noda H., Ohya H., Kamada H., Hirayama A., Koyama A. (2003). Importance of Renal Mitochondria in the Reduction of TEMPOL, a Nitroxide Radical. Mol. Cell. Biochem..

[39] Keana J.F., Pou S., Rosen G.M. (1987). Nitroxides as Potential Contrast Enhancing Agents for MRI Application: Influence of Structure on the Rate of Reduction by Rat Hepatocytes, Whole Liver Homogenate, Subcellular Fractions, and Ascorbate. Magn. Reson. Med..

[40] Iannone A., Tomasi A., Vannini V., Swartz H.M. (1990). Metabolism of Nitroxide Spin Labels in Subcellular Fraction of Rat Liver. Biochim. Biophys. Acta BBA Gen. Subj..

[41] Babić N., Orio M., Peyrot F. (2020). Unexpected Rapid Aerobic Transformation of 2,2,6,6-Tetraethyl-4-Oxo(Piperidin-1-Yloxyl) Radical by Cytochrome P450 in the Presence of NADPH: Evidence against a Simple Reduction of the Nitroxide Moiety to the Hydroxylamine. Free Radic. Biol. Med..

[42] Jagtap A.P., Krstic I., Kunjir N.C., Hänsel R., Prisner T.F., Sigurdsson S.T. (2015). Sterically Shielded Spin Labels for In-Cell EPR Spectroscopy: Analysis of Stability in Reducing Environment. Free Radic. Res..

[43] Kajer T.B., Fairfull-Smith K.E., Yamasaki T., Yamada K., Fu S., Bottle S.E., Hawkins C.L., Davies M.J. (2014). Inhibition of Myeloperoxidase- and Neutrophil-Mediated Oxidant Production by Tetraethyl and Tetramethyl Nitroxides. Free Radic. Biol. Med..

[44] Kinoshita Y., Yamada K., Yamasaki T., Mito F., Yamato M., Kosem N., Deguchi H., Shirahama C., Ito Y., Kitagawa K. (2010). In Vivo Evaluation of Novel Nitroxyl Radicals with Reduction Stability. Free Radic. Biol. Med..

[45] Griffiths P.G., Rizzardo E., Solomon D.H. (1982). Quantitative Studies on Free Radical Reactions with the Scavenger 1,1,3,3-Tetramethylisoindolinyl-2-Oxy. Tetrahedron Lett..

[46] Khan N., Blinco J.P., Bottle S.E., Hosokawa K., Swartz H.M., Micallef A.S. (2011). The Evaluation of New and Isotopically Labeled Isoindoline Nitroxides and an Azaphenalene Nitroxide for EPR Oximetry. J. Magn. Reson..

[47] Kuppusamy P., Chzhan M., Zweier J.L., Berliner L.J. (2003). Principles of Imaging: Theory and Instrumentation. In Vivo EPR (ESR).

[48] Wang Y., Paletta J.T., Berg K., Reinhart E., Rajca S., Rajca A. (2014). Synthesis of Unnatural Amino Acids Functionalized with Sterically Shielded Pyrroline Nitroxides. Org. Lett..

[49] Hatano B., Araya H., Yoshimura Y., Sato H., Ito T., Ogata T., Kijima T. (2010). Facile Synthesis of 3-Methoxycarbonyl-2,2,5,5-Tetra-Methylpyrrolidine-1-Oxyl and Derivatives. Heterocycles.

[50] Fairfull-Smith K.E., Brackmann F., Bottle S.E. (2009). The Synthesis of Novel Isoindoline Nitroxides Bearing Water-Solubilising Functionality. Eur. J. Org. Chem..

[51] Smith C.D., Bartley J.P., Bottle S.E., Micallef A.S., Reid D.A. (2000). Electrospray Ionization Mass Spectrometry of Stable Nitroxide Free Radicals and Two Isoindoline Nitroxide Dimers. J. Mass Spectrom..

[52] Scherrer R.A., Howard S.M. (1977). Use of Distribution Coefficients in Quantitative Structure-Activity Relations. J. Med. Chem..

[53] Kremers P., Beaune P., Cresteil T., Graeve J., Columelli S., Leroux J.-P., Gielen J.E. (1981). Cytochrome P-450 Monooxygenase Activities in Human and Rat Liver Microsomes. Eur. J. Biochem..

[54] Bradford M.M. (1976). A Rapid and Sensitive Method for the Quantitation of Microgram Quantities of Protein Utilizing the Principle of Protein-Dye Binding. Anal. Biochem..

[55] Vermilion J.L., Coon M.J. (1978). Purified Liver Microsomal NADPH-Cytochrome P-450 Reductase. Spectral Characterization of Oxidation-Reduction States. J. Biol. Chem..

[56] Omura T., Sato R. (1964). The Carbon Monoxide-Binding Pigment of Liver Microsomes. J. Biol. Chem..

[57] Bézière N., Hardy M., Poulhès F., Karoui H., Tordo P., Ouari O., Frapart Y.-M., Rockenbauer A., Boucher J.-L., Mansuy D. (2014). Metabolic Stability of Superoxide Adducts Derived from Newly Developed Cyclic Nitrone Spin Traps. Free Radic. Biol. Med..

[58] Babić N., Peyrot F. (2019). New Synthetic Route to 2,2,6,6-Tetraethylpiperidin-4-One: A Key-Intermediate towards Tetraethyl Nitroxides. Tetrahedron Lett..

[59] Stoll S., Schweiger A. (2006). EasySpin, a Comprehensive Software Package for Spectral Simulation and Analysis in EPR. J. Magn. Reson..

[60] Neese F. (2012). The ORCA Program System. WIREs Comput. Mol. Sci..

[61] Becke A.D. (1993). A New Mixing of Hartree–Fock and Local Density-functional Theories. J. Chem. Phys..

[62] Lee C., Yang W., Parr R.G. (1988). Development of the Colle-Salvetti Correlation-Energy Formula into a Functional of the Electron Density. Phys. Rev. B.

[63] Csonka G.I. (2002). Proper Basis Set for Quantum Mechanical Studies of Potential Energy Surfaces of Carbohydrates. J. Mol. Struct..

[64] Boese A.D., Martin J.M.L., Handy N.C. (2003). The Role of the Basis Set: Assessing Density Functional Theory. J. Chem. Phys..

[65] Mackie I.D., DiLabio G.A. (2010). Accurate Dispersion Interactions from Standard Density-Functional Theory Methods with Small Basis Sets. Phys. Chem. Chem. Phys..

[66] Ho J., Ertem M.Z. (2016). Calculating Free Energy Changes in Continuum Solvation Models. J. Phys. Chem. B.

[67] Franchi P., Lucarini M., Pedrielli P., Pedulli G.F. (2002). Nitroxide Radicals as Hydrogen Bonding Acceptors. An Infrared and EPR Study. ChemPhysChem.

[68] Owenius R., Engström M., Lindgren M., Huber M. (2001). Influence of Solvent Polarity and Hydrogen Bonding on the EPR Parameters of a Nitroxide Spin Label Studied by 9-GHz and 95-GHz EPR Spectroscopy and DFT Calculations. J. Phys. Chem. A.

[69] Hermosilla L., Vega J.G.D.L., Sieiro C., Calle P. (2011). DFT Calculations of Isotropic Hyperfine Coupling Constants of Nitrogen Aromatic Radicals: The Challenge of Nitroxide Radicals. J. Chem. Theory Comput..

[70] Dikalov S., Skatchkov M., Bassenge E. (1997). Spin Trapping of Superoxide Radicals and Peroxynitrite by 1-Hydroxy-3-Carboxy-Pyrrolidine and 1-Hydroxy-2,2,6,6-Tetramethyl-4-Oxo-Piperidine and the Stability of Corresponding Nitroxyl Radicals Towards Biological Reductants. Biochem. Biophys. Res. Commun..

[71] Huang S., Zhang H., Paletta J.T., Rajca S., Rajca A. (2018). Reduction Kinetics and Electrochemistry of Tetracarboxylate Nitroxides. Free Radic. Res..

[72] Tew D.G. (1993). Inhibition of Cytochrome P450 Reductase by the Diphenyliodonium Cation. Kinetic Analysis and Covalent Modifications. Biochemistry.

[73] Crowley P.D., Gallagher H.C. (2014). Clotrimazole as a Pharmaceutical: Past, Present and Future. J. Appl. Microbiol..

[74] Lewis D.F.V., Pratt J.M. (1998). The P450 Catalytic Cycle and Oxygenation Mechanism. Drug Metab. Rev..

[75] Amunom I., Srivastava S., Prough R.A. (2011). Aldehyde Reduction by Cytochrome P450. Curr. Protoc. Toxicol..

[76] Guengerich F.P. (1983). Oxidation-Reduction Properties of Rat Liver Cytochromes P-450 and NADPH-Cytochrome P-450 Reductase Related to Catalysis in Reconstituted Systems. Biochemistry.

[77] Yamazaki H., Johnson W.W., Ueng Y.-F., Shimada T., Guengerich F.P. (1996). Lack of Electron Transfer from Cytochrome B5 in Stimulation of Catalytic Activities of Cytochrome P450 3A4: Characterization of a Reconstituted Cytochrome P450 3A4/NADPH-Cytochrome P450 Reductase System and Studies With APO-Cytochrome B5*. J. Biol. Chem..

[78] Correia M.A., Ortiz de Montellano P.R., Ortiz de Montellano P.R. (2005). Inhibition of Cytochrome P450 Enzymes. Cytochrome P450: Structure, Mechanism, and Biochemistry.

[79] Guengerich F.P. (2001). Common and Uncommon Cytochrome P450 Reactions Related to Metabolism and Chemical Toxicity. Chem. Res. Toxicol..

[80] Lewis D.F.V., Jacobs M.N., Dickins M. (2004). Compound Lipophilicity for Substrate Binding to Human P450s in Drug Metabolism. Drug Discov. Today.

[81] Mehlhorn R.J., Swanson C.E. (1992). Nitroxide-Stimulated H2O2 Decomposition by Peroxidases and Pseudoperoxidases. Free Radic. Res. Commun..

[82] Krishna M.C., Samuni A., Taira J., Goldstein S., Mitchell J.B., Russo A. (1996). Stimulation by Nitroxides of Catalase-like Activity of Hemeproteins: Kinetics and Mechanism. J. Biol. Chem..

[83] Yamaguchi T., Nakano T., Kimoto E. (1984). Oxidation of Nitroxide Radicals by the Reaction of Hemoglobin with Hydrogen Peroxide. Biochem. Biophys. Res. Commun..

[84] Bono D.D., Yang W.-D., Symons M.C.R. (1994). The Effect of Myoglobin on the Stability of the Hydroxyl-Radical Adducts of 5, 5 Dimethyl-1-Pyrolline-N-Oxide (DMPO), 3, 3,5, 5 Tetramethyl-1-Pyrolline-N-Oxide(TMPO) and 1-Alpha-Phenyl-Tert-Butyl Nitrone (PBN) in the Presence of Hydrogen Peroxide. Free Radic. Res..

[85] Kroll C., Borchert H.-H. (1999). Metabolism of the Stable Nitroxyl Radical 4-Oxo-2,2,6,6-Tetramethylpiperidine-N-Oxyl (TEMPONE). Eur. J. Pharm. Sci..

[86] Isin E.M., Guengerich F.P. (2007). Complex Reactions Catalyzed by Cytochrome P450 Enzymes. Biochim. Biophys. Acta BBA Gen. Subj..

[87] Mittra K., Green M.T. (2019). Reduction Potentials of P450 Compounds I and II: Insight into the Thermodynamics of C–H Bond Activation. J. Am. Chem. Soc..

[88] Guengerich F.P. (2018). Mechanisms of Cytochrome P450-Catalyzed Oxidations. ACS Catal..

[89] Parmentier Y., Bossant M.-J., Bertrand M., Walther B., Taylor J.B., Triggle D.J. (2007). 5.10—In Vitro Studies of Drug Metabolism. Comprehensive Medicinal Chemistry II.

[90] Iannone A., Bini A., Swartz H.M., Tomasi A., Vannini V. (1989). Metabolism in Rat Liver Microsomes of the Nitroxide Spin Probe Tempol. Biochem. Pharmacol..

[91] Zhitkovich A. (2020). Nuclear and Cytoplasmic Functions of Vitamin C. Chem. Res. Toxicol..

[92] Hoch M.J.R., Ewert U. (1983). Resolution in EPR Imaging*. EPR Imaging and In Vivo EPR.

[93] Valvis I.I., Lischick D., Shen D., Sofer S.S. (1990). In Vitro Synthesis of Nitroxide Free Radicals by Hog Liver Microsomes. Free Radic. Biol. Med..

[94] Olsen L., Rydberg P., Rod T.H., Ryde U. (2006). Prediction of Activation Energies for Hydrogen Abstraction by Cytochrome P450. J. Med. Chem..

