# Supplementary material for: Structural Features Governing the Metabolic Stability of Tetraethyl-Substituted Nitroxides in Rat Liver Microsomes
Source: Antioxidants (Basel). 2023 Feb 7;12(2):402. doi: 10.3390/antiox12020402 (PMC9952648; doi:10.3390/antiox12020402)
Supplement: Supplementary file 1 [file antioxidants-12-00402-s001.zip › antioxidants-2155840-supplementary.pdf]

Supplementary material

Structural features governing the metabolic stability of tetraethyl-substituted nitroxides in rat liver microsomes

A. Rančić [1], N. Babić [1], M. Orio [2], F. Peyrot [1,3]

(1) Université Paris Cité, CNRS, Laboratoire de Chimie et de Biochimie Pharmacologiques et Toxicologiques, F-75006 Paris, France; (2) Aix-Marseille Univ., CNRS, Centrale Marseille, iSm2; (3) Sorbonne Université, Institut National Supérieur du Professorat et de l'Education (INSPE) de l'Académie de Paris, Paris, France

Table of contents

1. Estimation of lipophilicity 1
2. Characterization of nitroxide **5** 1
3. HPLC-HRMS analysis of anaerobic incubations of nitroxide **5** with ascorbate 3
4. EasySpin simulations 4
5. HPLC-HRMS analysis of nitroxide **5** when incubated with RLM enriched in NADPH under anaerobic and aerobic conditions 5
6. DFT calculations 6

1. Estimation of lipophilicity

Supplementary Table S1. $ClogP$, pKa and $logD_{7.4}$ values calculated for ionizable compounds under study.

Nitroxide	**2**	**3**	**4**	**5**
$ClogP$	0.811	2.927	2.828	4.261
pKa	4.776	4.835	3.519	4.434
$logD_{7.4}$	−1.814	0.362	−1.054	1.295

2. Characterization of nitroxide 5

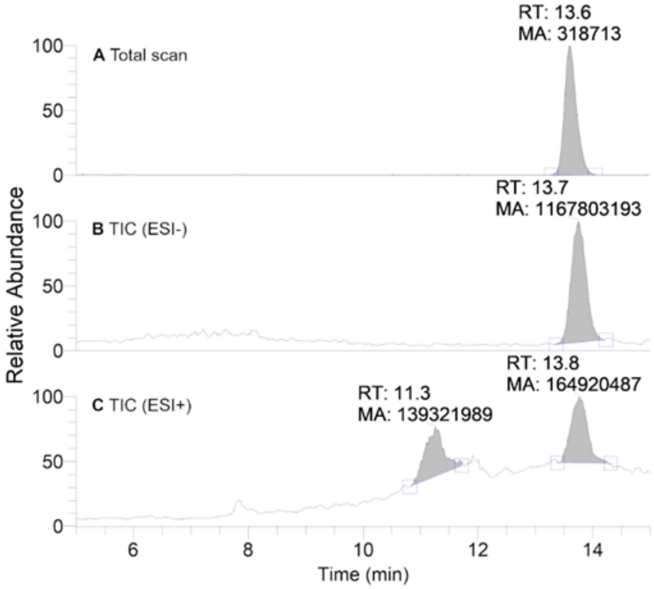

Supplementary Figure S1. Total scan UV-Vis (**A**), TIC (ESI^-) (**B**) and (ESI^+) (**C**) chromatograms of nitroxide **5** (100 µM).

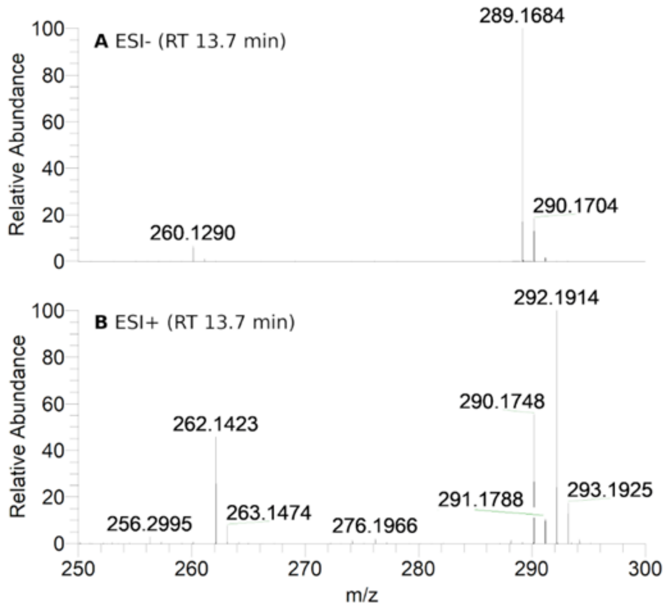

Supplementary Figure S2. HRMS spectrum of nitroxide **5** in positive (**A**) and negative mode (**B**). m/z calcd. for $C_{17}H_{23}NO_3$ [(M−H)$^-$]: 289.1683; $C_{17}H_{24}NO_3$ [M^+]: 290.1751; $C_{17}H_{26}NO_3$ [(M+$2H$)$^+$]: 292.1907. Fragments at m/z calcd. for $C_{15}H_{18}NO_3$ [(M−C_2H_5−H)$^-$]: 260.1292; $C_{15}H_{20}NO_3$ [(M−C_2H_5+H)$^+$]: 262.1437 are consistent with fragmentation patterns observed for other isoindoline nitroxides [1].

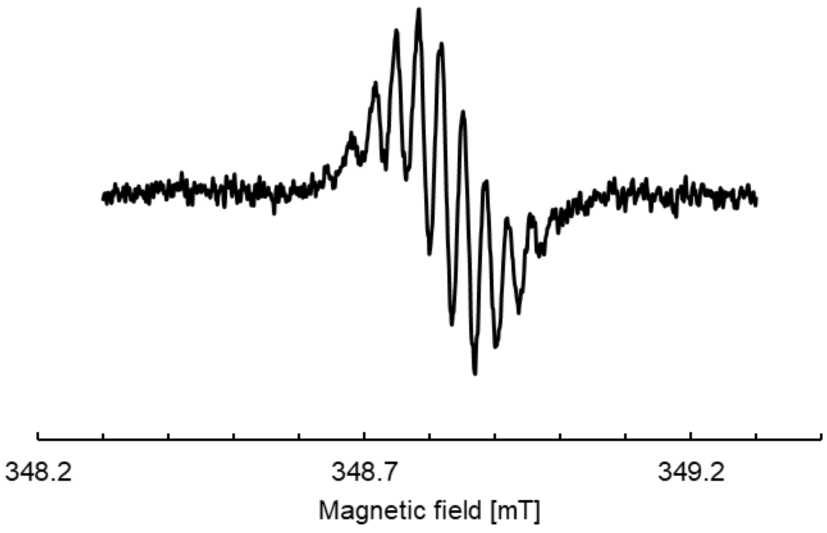

Supplementary Figure S3. Superhyperfine structure of nitroxide **5** (100 µM) in potassium phosphate buffer (0.1 M, pH 7.4, containing 1 mM DTPA) in the absence of oxygen at 21 °C: close-up on the low-field line. The recording parameters are given in the experimental section.

3. HPLC-HRMS analysis of anaerobic incubations of nitroxide 5 with ascorbate

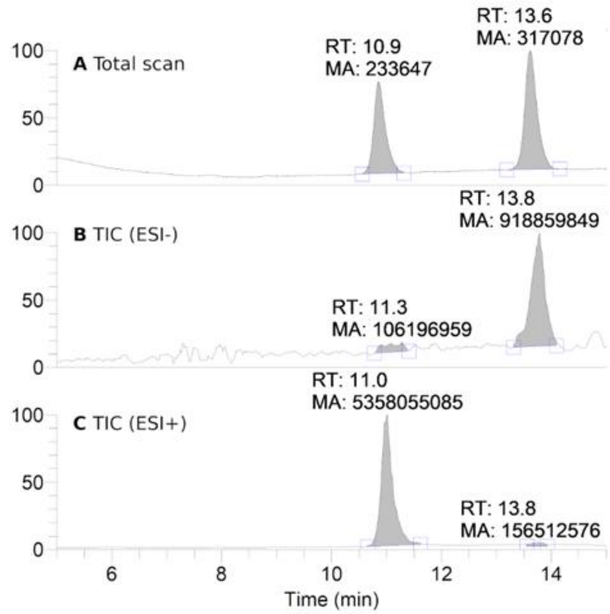

Supplementary Figure S4. Total scan UV-Vis (A), TIC (ESI^-) (**B**) and (ESI^+) (**C**) chromatograms of the incubations of nitroxide **5** (100 µM) with sodium L-ascorbate (800 mM) in pure water upon anaerobic conditions. Samples were prepared and analyzed as described in the experimental section.

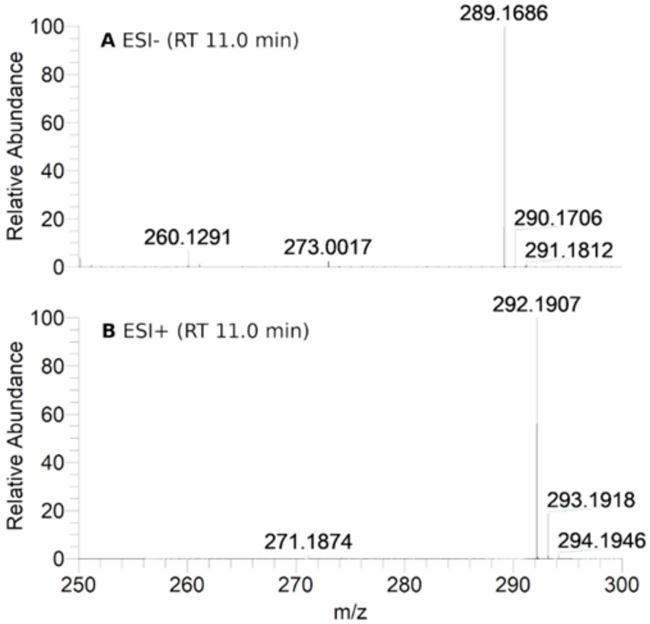

Supplementary Figure S5. HRMS spectra of the hydroxylamine product eluted at RT 11.0 min in the HPLC analysis of the anaerobic incubation of nitroxide **5** (100 µM) with sodium L-ascorbate (800 mM) in pure water after 60 min obtained in ESI^- (**A**) and ESI^+ (**B**) modes, respectively.

4. EasySpin simulations

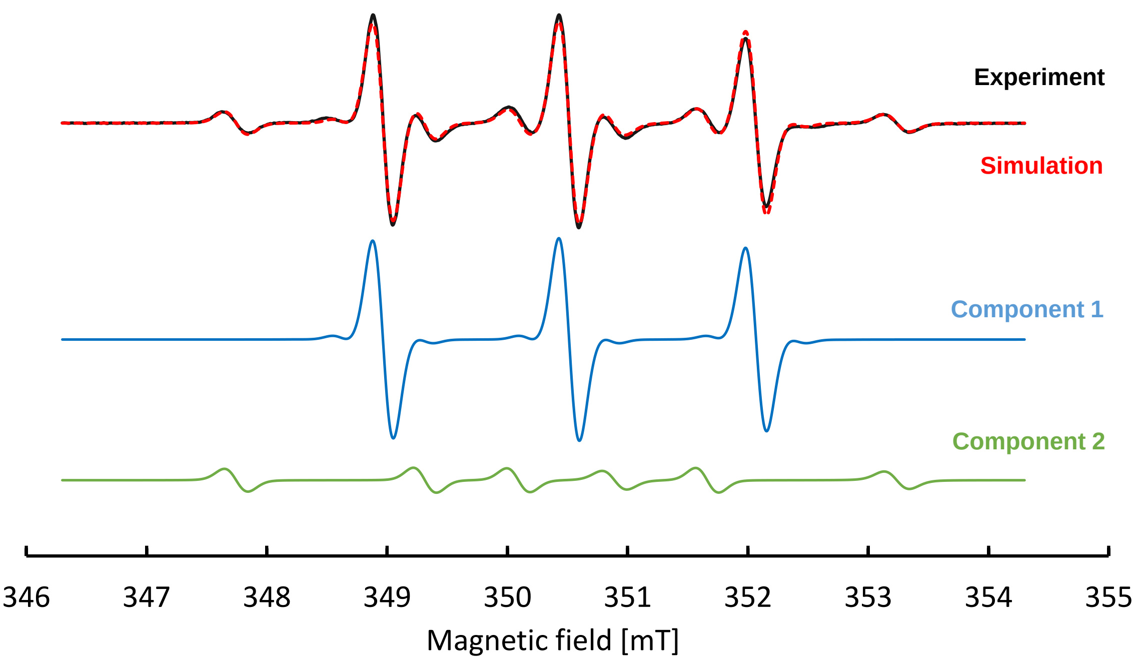

Supplementary Figure S6. Experimental spectrum (black line) of nitroxide **5** (300 µM) incubated with RLM (7.8 mg·ml^{-1} protein, 30 µM P450) enriched in 2 mM NADPH after 10 min under aerobic conditions at 21 °C overlapped with the simulated EPR spectrum (red dashed line). Below are the simulated spectra of nitroxide **5** (component 1, blue line) and the simulated EPR spectrum of the new 6-line species (component 2, green line). The parameters used in the simulation are given in Supplementary Table S2.

Supplementary Table S2. EPR spectral characteristics of identified species observed in the reaction of nitroxide **5** with RLM and NADPH. The hyperfine coupling constants (A_N, A_H, $A_{^{13}C}$) and linewidth parameter (ΔB) were extracted by simulation with EasySpin. The concentrations (C [µM]) are calculated from the ratio of the two compounds extracted from the simulation and a calibration curve for nitroxide **5**. Presented values estimate the concentrations of nitroxide **5** and 6-line intermediate 10 minutes after mixing 300 µM nitroxide **5** with RLM (7.7 mg prot·ml^{-1}; 30 µM P450) and 2 mM NADPH upon aerobic conditions at 21 °C.

	Nitroxide **5**	6-line intermediate
A_N [mT]/[MHz]	1.56/43.6	15.8/44.3
A_H [mT]/[MHz]	-	2.36/66.0
$A_{^{13}C}$ [mT]/[MHz]	0.68/19.0	-
ΔB [mT]	0.19	0.21

5. HPLC-HRMS analysis of nitroxide 5 when incubated with RLM enriched in NADPH under anaerobic and aerobic conditions

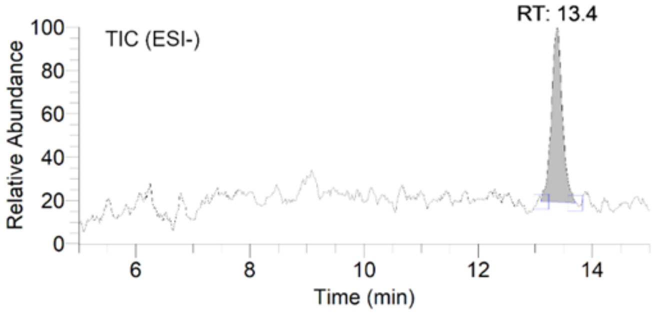

Supplementary Figure S7. HPLC-HRMS study of nitroxide **5** (300 µM) incubated with RLM (7.8 mg·ml^{-1} protein, 30 µM P450) supplemented with NADPH (2 mM) in potassium phosphate buffer (100 mM; pH 7.4) upon aerobic conditions after 60 minutes: the TIC chromatogram in ESI^{-} only shows the starting material at RT = 13.4 min. Peaks in TIC chromatograms are observed with a delay of 0.2 min compared to UV-Vis detection.

Supplementary Table S3. Main absorption bands in the UV spectra from HPLC-HRMS study of nitroxide **5** (300 µM) incubated with RLM (7.8 mg ml^{-1} protein, 30 µM P450) enriched in NADPH (2 mM) in potassium phosphate buffer (0.1 M, pH 7.4) under aerobic conditions at 21°C (see Figure 6).

RT [min]	UV absorption bands [nm]
6.0	233
7.6	312
7.9	273
8.8	239
9.0	272
9.3	239
13.2	237

6. DFT calculations

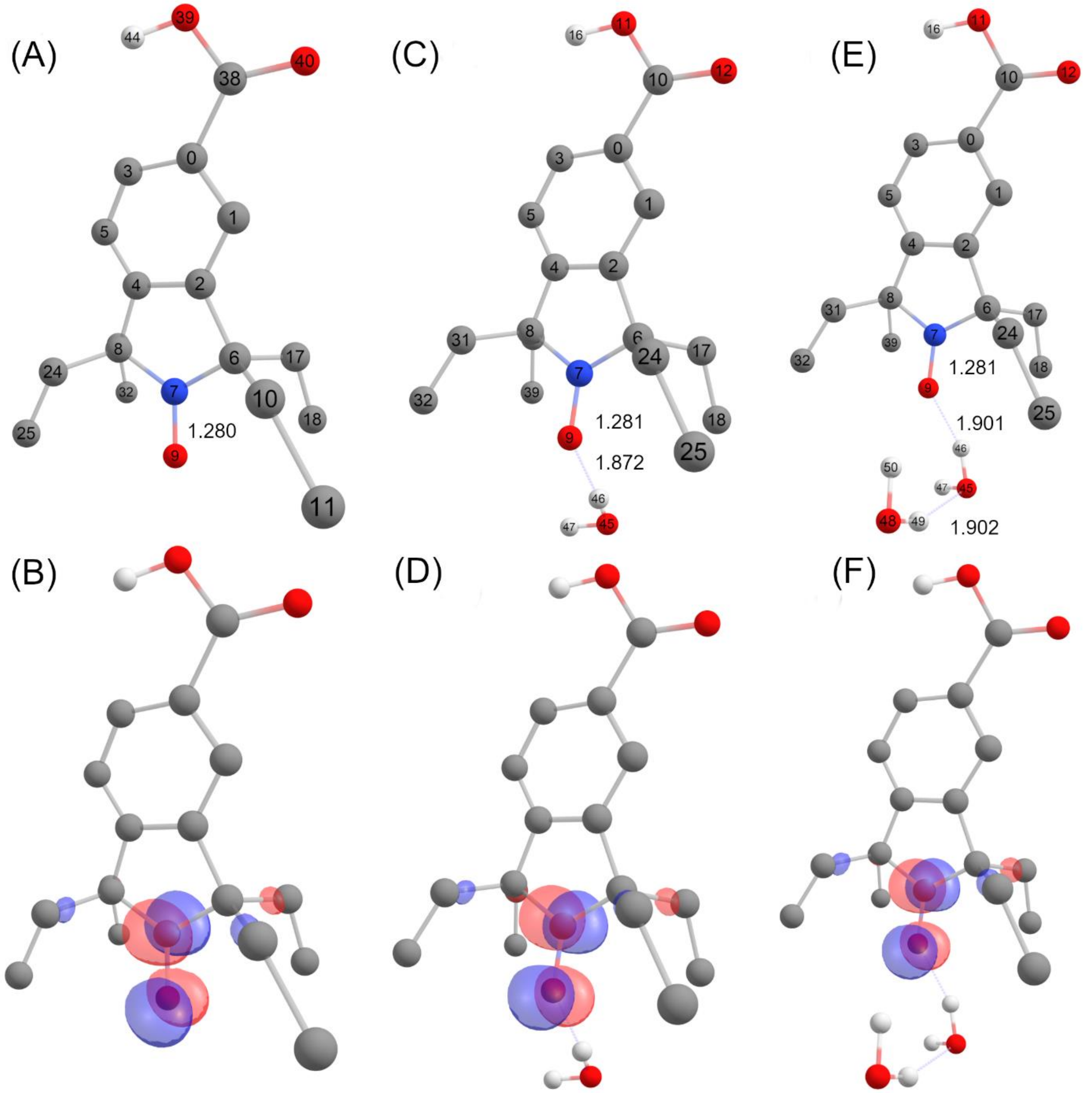

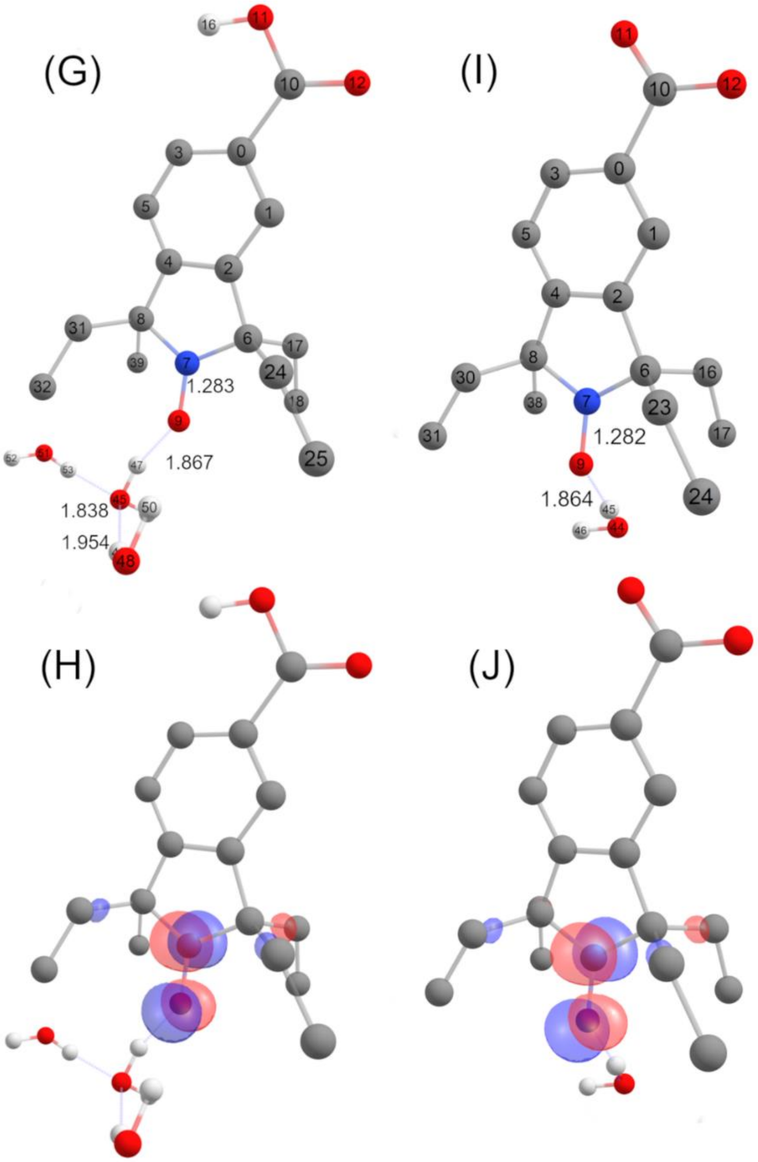

Supplementary Figure S8. DFT-optimized structures for nitroxide **5** without (**A**, **B**), with one (**C**, **D**), two (**E**, **F**), and three (**G**, **H**) explicit water molecules and in carboxylate form with one explicit water molecule (**I**, **J**) together with selected metrical parameters (Å) (**A**, **C**, **E**, **G**, **I**), and localized SOMOs (**B**, **D**, **F**, **H**, **J**). Hydrogen atoms attached to carbon centers were omitted of clarity.

Supplementary Table S4. DFT-computed hyperfine coupling constants of nitroxide **5** using the B3LYP/G functional and various basis sets.

Basis set	A_N [MHz]	A_N [G]
TZVP	25.82	9.21
EPR-II	28.12	10.03
SVP	58.07	20.72
6-31g*	**37.19**	**13.27**
EPR-III	Not converged	

Supplementary Table S5. Free energies and DFT-computed nitrogen hyperfine coupling constants of nitroxide **5** and models including one, two and three explicit water molecules.

Model	Gibbs free energy [Eh]	A_N [MHz]
**5** (carboxylic acid form)	−942.301962	37.27
**5** (carboxylic acid form)+H_2O	−1018.705870	41.76
**5** (carboxylic acid form)+2H_2O	−1095.113281	41.76
**5** (carboxylic acid form)+3H_2O	−1171.519225	40.92
**5** (carboxylate form)+H_2O	−1018.596155	42.06

Supplementary Table S6. Free energies of other structures considered with and without one explicit water molecule.

Model	Gibbs free energy [Eh]	A_N [MHz]	A_H [MHz]
**A**	−862.810071	28.52	28.91
**A** + H_2O	−939.236803	30.18	29.85
**B**	−941.412933	47.83	9.92
**B** + H_2O	−1017.838986	40.58	48.23
**C** *cis*	−1017.842710	37.34	13.32
**C** *cis* + H_2O	−1094.274882	40.08	5.95
**D**	−941.420185	31.48	15.20
**D** + H_2O	−1017.845624	34.45	14.85
**E**	−1017.855334	39.36	3.46
**E** + H_2O	−1094.286646	48.18	1.33

Supplementary Table S7. DFT-optimized Cartesian coordinates for nitroxide **5**

6	−3.955027000	1.890439000	−1.037488000
6	−2.776848000	2.540697000	−0.636745000
6	−1.667646000	1.784811000	−0.276111000
6	−3.994285000	0.486898000	−1.095108000
6	−1.713944000	0.387607000	−0.330248000
6	−2.873788000	−0.267026000	−0.745548000
6	−0.316225000	2.293406000	0.191558000
7	0.391872000	0.990590000	0.392347000
6	−0.400488000	−0.241723000	0.090364000

8	1.539587000	0.929578000	0.956130000
6	−0.496053000	3.051166000	1.539757000
6	0.728452000	3.708496000	2.187697000
1	−0.945821000	2.344264000	2.248683000
1	−1.254163000	3.824375000	1.358798000
1	1.538506000	2.989617000	2.330245000
1	0.444523000	4.109147000	3.167639000
1	1.104313000	4.545178000	1.590193000
6	0.350190000	3.139562000	−0.937261000
6	1.879708000	3.270215000	−0.947997000
1	−0.107081000	4.137047000	−0.896846000
1	0.044503000	2.704801000	−1.896862000
1	2.275714000	3.745235000	−0.047763000
1	2.174159000	3.883736000	−1.807871000
1	2.362385000	2.294846000	−1.049048000
6	−0.633315000	−1.092959000	1.373869000
6	0.547990000	−1.870145000	1.965430000
1	−1.434739000	−1.801971000	1.130779000
1	−1.043555000	−0.419668000	2.137220000
1	0.876347000	−2.677927000	1.303347000
1	0.239141000	−2.328941000	2.911884000
1	1.397550000	−1.212604000	2.160148000
6	0.209667000	−1.039475000	−1.104157000
6	1.729250000	−1.254465000	−1.136534000
1	−0.075361000	−0.519576000	−2.027015000
1	−0.302912000	−2.009810000	−1.130856000
1	1.984127000	−1.825011000	−2.037859000
1	2.101864000	−1.809492000	−0.272682000
1	2.265012000	−0.302963000	−1.177442000
6	−5.117893000	2.744074000	−1.419188000
8	−6.342667000	2.171957000	−1.437722000
8	−5.018326000	3.922299000	−1.712090000
1	−2.757976000	3.625527000	−0.608552000
1	−4.882261000	−0.034762000	−1.440957000
1	−2.914470000	−1.350897000	−0.804805000
1	−6.322498000	1.272994000	−1.065623000

Supplementary Table S8. DFT-optimized Cartesian coordinates for model **5** + H_2O

6	−4.768361000	1.327030000	−1.455512000
6	−3.729713000	2.031223000	−0.826509000
6	−2.667175000	1.328996000	−0.269421000
6	−4.712202000	−0.074895000	−1.537377000
6	−2.619914000	−0.065727000	−0.347732000
6	−3.637987000	−0.773779000	−0.987084000
6	−1.461047000	1.904392000	0.449550000
7	−0.711809000	0.638541000	0.749712000
6	−1.376043000	−0.633120000	0.308860000
8	0.245452000	0.619580000	1.601001000

6	−5.883306000	2.125957000	−2.044498000
8	−7.036489000	1.484865000	−2.336542000
8	−5.804075000	3.319847000	−2.273522000
1	−3.781139000	3.114609000	−0.780568000
1	−5.483844000	−0.638532000	−2.054707000
1	−3.602636000	−1.856552000	−1.066666000
1	−7.032883000	0.571487000	−2.001286000
6	−0.667031000	2.833425000	−0.518750000
6	0.824308000	3.072976000	−0.250787000
1	−1.198448000	3.793572000	−0.533006000
1	−0.764862000	2.417665000	−1.528827000
1	1.019443000	3.498229000	0.735785000
1	1.206292000	3.777588000	−0.999140000
1	1.405962000	2.152334000	−0.338576000
6	−1.933110000	2.598068000	1.762485000
6	−0.911935000	3.371833000	2.604433000
1	−2.407120000	1.826754000	2.382107000
1	−2.735511000	3.286339000	1.468307000
1	−0.063799000	2.743518000	2.884853000
1	−1.397759000	3.723414000	3.521887000
1	−0.534509000	4.253564000	2.077374000
6	−1.802141000	−1.497318000	1.534153000
6	−0.737538000	−2.291100000	2.300325000
1	−2.559312000	−2.200039000	1.163691000
1	−2.325647000	−0.829042000	2.229077000
1	−0.326970000	−3.108105000	1.699146000
1	−1.199191000	−2.741081000	3.186899000
1	0.084250000	−1.651512000	2.629343000
6	−0.523014000	−1.381327000	−0.759953000
6	0.967727000	−1.620370000	−0.484870000
1	−0.606833000	−0.815496000	−1.695358000
1	−1.022385000	−2.342321000	−0.940847000
1	1.403866000	−2.131276000	−1.351776000
1	1.144228000	−2.247217000	0.391675000
1	1.515820000	−0.686245000	−0.342445000
8	3.048662000	0.776592000	1.151689000
1	2.088438000	0.808571000	1.331956000
1	3.276455000	−0.151253000	1.321273000

Supplementary Table S9. DFT-optimized Cartesian coordinates for model **5** + 2H_2O

6	−4.819507000	1.340888000	−1.289623000
6	−3.719891000	2.051400000	−0.783514000
6	−2.619263000	1.352647000	−0.301518000
6	−4.786799000	−0.063361000	−1.329911000
6	−2.594856000	−0.045174000	−0.337203000
6	−3.674109000	−0.759449000	−0.857562000
6	−1.348166000	1.934209000	0.290993000
7	−0.603758000	0.666910000	0.579313000

6	−1.303529000	−0.607112000	0.224042000
8	0.447847000	0.651522000	1.309956000
6	−5.977780000	2.134079000	−1.797230000
8	−7.165241000	1.502980000	−1.932076000
8	−5.904347000	3.313355000	−2.093036000
1	−3.755131000	3.136279000	−0.770247000
1	−5.609987000	−0.630580000	−1.755131000
1	−3.657910000	−1.844617000	−0.902872000
1	−7.140929000	0.608753000	−1.548981000
6	−0.613425000	2.810079000	−0.771659000
6	0.909890000	2.959821000	−0.665027000
1	−1.086272000	3.799834000	−0.740431000
1	−0.841470000	2.395493000	−1.760618000
1	1.238843000	3.361288000	0.296269000
1	1.252910000	3.645518000	−1.448242000
1	1.416647000	2.003355000	−0.822701000
6	−1.696794000	2.694339000	1.605855000
6	−0.595861000	3.463917000	2.344934000
1	−2.159194000	1.971037000	2.289417000
1	−2.488847000	3.403186000	1.334229000
1	0.209789000	2.810597000	2.687356000
1	−1.035033000	3.943431000	3.227617000
1	−0.166713000	4.258202000	1.726129000
6	−1.617173000	−1.447959000	1.498132000
6	−0.482351000	−2.221750000	2.179515000
1	−2.393208000	−2.163917000	1.200699000
1	−2.088825000	−0.774254000	2.224602000
1	−0.073553000	−3.001340000	1.528740000
1	−0.880257000	−2.719485000	3.071523000
1	0.334178000	−1.567027000	2.492053000
6	−0.532317000	−1.390653000	−0.883997000
6	0.993787000	−1.502516000	−0.763528000
1	−0.761640000	−0.917956000	−1.846307000
1	−0.976075000	−2.393683000	−0.923750000
1	1.369094000	−2.100845000	−1.601933000
1	1.317067000	−1.982232000	0.162640000
1	1.472788000	−0.521027000	−0.815067000
8	3.070343000	1.545213000	1.794172000
1	2.220846000	1.335055000	1.356785000
1	3.628136000	0.770161000	1.613465000
8	1.482180000	0.739551000	3.998299000
1	2.210338000	1.046151000	3.416036000
1	0.771498000	0.603605000	3.348865000

Supplementary Table S10. DFT-optimized Cartesian coordinates for model **5** + 3H_2O

6	−4.766622000	1.014289000	−1.203814000
6	−3.790085000	1.884066000	−0.693151000
6	−2.580539000	1.365556000	−0.246083000

6	−4.503943000	−0.363718000	−1.281545000
6	−2.326311000	−0.007550000	−0.322271000
6	−3.282784000	−0.877654000	−0.844760000
6	−1.403422000	2.134174000	0.326327000
7	−0.479805000	0.993996000	0.620857000
6	−0.945334000	−0.364085000	0.198442000
8	0.581085000	1.176534000	1.318148000
6	−6.046480000	1.619565000	−1.677863000
8	−7.113274000	0.804555000	−1.831394000
8	−6.173573000	2.804352000	−1.930570000
1	−4.003143000	2.947268000	−0.652308000
1	−5.229001000	−1.050322000	−1.709390000
1	−3.089361000	−1.943354000	−0.921436000
1	−6.934633000	−0.087878000	−1.486724000
6	−0.809081000	3.078946000	−0.766373000
6	0.673003000	3.464484000	−0.667093000
1	−1.429942000	3.983616000	−0.767672000
1	−0.968071000	2.600527000	−1.740168000
1	0.925190000	3.963213000	0.270827000
1	0.912921000	4.150016000	−1.488557000
1	1.321049000	2.589186000	−0.764333000
6	−1.830351000	2.862348000	1.635110000
6	−0.861937000	3.857399000	2.286493000
1	−2.109640000	2.089637000	2.361775000
1	−2.756338000	3.399351000	1.393052000
1	0.090224000	3.397051000	2.559828000
1	−1.321273000	4.249641000	3.200967000
1	−0.657025000	4.710748000	1.632930000
6	−1.108730000	−1.330645000	1.409856000
6	0.106697000	−1.745817000	2.248017000
1	−1.573625000	−2.233725000	0.995168000
1	−1.856892000	−0.885736000	2.077861000
1	0.907442000	−2.192757000	1.653698000
1	−0.218814000	−2.499423000	2.975528000
1	0.522659000	−0.908069000	2.812373000
6	−0.090364000	−0.924612000	−0.983992000
6	1.439203000	−0.861804000	−0.902567000
1	−0.398306000	−0.382474000	−1.885595000
1	−0.410206000	−1.964141000	−1.130890000
1	1.850470000	−1.230965000	−1.849631000
1	1.865894000	−1.472266000	−0.104699000
1	1.792339000	0.165727000	−0.775216000
8	3.076186000	0.198384000	2.096560000
1	3.728067000	0.821923000	1.733751000
1	2.247094000	0.383140000	1.603814000
8	1.370269000	1.315266000	4.100253000
1	2.162477000	0.967053000	3.643460000
1	0.747551000	1.397906000	3.357544000

8	3.615272000	−2.501225000	1.499193000
1	3.229278000	−2.959749000	2.261863000
1	3.494315000	−1.551085000	1.719221000

Supplementary Table S11. DFT-optimized Cartesian coordinates for model **5** (carboxylate form) + H_2O

6	−4.775786000	1.339695000	−1.464412000
6	−3.740368000	2.031569000	−0.822906000
6	−2.671917000	1.328456000	−0.267773000
6	−4.716713000	−0.060615000	−1.534672000
6	−2.623710000	−0.065226000	−0.344208000
6	−3.649061000	−0.769187000	−0.980105000
6	−1.461702000	1.903252000	0.447116000
7	−0.711293000	0.638543000	0.749028000
6	−1.377574000	−0.633107000	0.310765000
8	0.249297000	0.620636000	1.598533000
6	−5.952357000	2.102879000	−2.082660000
8	−6.843203000	1.409387000	−2.646959000
8	−5.929486000	3.360385000	−1.975427000
1	−3.799194000	3.115203000	−0.773412000
1	−5.527367000	−0.581335000	−2.034511000
1	−3.620683000	−1.854678000	−1.046081000
6	−0.666141000	2.829648000	−0.522059000
6	0.826107000	3.069054000	−0.257672000
1	−1.196696000	3.790376000	−0.538913000
1	−0.766181000	2.411625000	−1.531047000
1	1.023643000	3.496705000	0.727530000
1	1.209582000	3.770616000	−1.008479000
1	1.405840000	2.146847000	−0.342402000
6	−1.925947000	2.601167000	1.760251000
6	−0.902177000	3.374541000	2.599886000
1	−2.400516000	1.832137000	2.382575000
1	−2.728080000	3.289864000	1.466315000
1	−0.053746000	2.745276000	2.877565000
1	−1.383923000	3.728010000	3.519043000
1	−0.524436000	4.255556000	2.071482000
6	−1.795934000	−1.495517000	1.539422000
6	−0.730230000	−2.288291000	2.305723000
1	−2.554857000	−2.198512000	1.172860000
1	−2.317960000	−0.826046000	2.234575000
1	−0.321620000	−3.107700000	1.706129000
1	−1.188012000	−2.735461000	3.195990000
1	0.093501000	−1.648157000	2.629100000
6	−0.523242000	−1.383409000	−0.755039000
6	0.967894000	−1.624578000	−0.482658000
1	−0.607741000	−0.818385000	−1.690918000
1	−1.023555000	−2.344168000	−0.936041000
1	1.404335000	−2.136563000	−1.349069000

1	1.145561000	−2.250461000	0.394540000
1	1.516082000	−0.690376000	−0.340725000
8	3.046656000	0.770435000	1.152136000
1	2.085452000	0.801382000	1.330633000
1	3.274209000	−0.157370000	1.322212000

Supplementary Table S12. DFT-optimized Cartesian coordinates for structure **C** *trans*

6	−4.164366000	2.009902000	−0.401543000
6	−3.107455000	2.917246000	−0.274984000
6	−1.936495000	2.603167000	0.421946000
6	−4.043236000	0.747467000	0.187531000
6	−1.802547000	1.314119000	0.986183000
6	−2.874244000	0.415631000	0.865088000
1	−3.231462000	3.894039000	−0.725759000
1	−2.817716000	−0.570932000	1.306118000
1	−4.825443000	−0.002225000	0.107419000
6	−5.362021000	2.443079000	−1.180953000
8	−5.363781000	3.406730000	−1.926161000
8	−6.496646000	1.722728000	−1.049518000
1	−6.414836000	1.046172000	−0.354818000
6	−0.915256000	3.724129000	0.649211000
6	−0.528353000	0.958798000	1.774543000
7	0.391797000	3.156145000	1.082112000
6	0.664744000	1.701405000	1.119843000
6	−0.218000000	−0.572799000	1.815177000
6	0.340620000	−1.090954000	3.148756000
1	0.481784000	−0.802542000	1.002567000
1	−1.111172000	−1.157662000	1.592572000
1	1.256215000	−0.578928000	3.454032000
1	0.565909000	−2.159149000	3.053566000
1	−0.389318000	−0.975037000	3.956469000
8	−0.657838000	1.510411000	3.098124000
1	−1.475406000	1.159073000	3.488509000
8	1.387846000	3.966807000	1.140314000
6	2.019697000	1.462260000	1.785818000
1	0.717149000	1.341644000	0.080798000
1	2.787646000	2.045309000	1.277274000
1	2.290441000	0.406857000	1.717665000
1	1.998274000	1.759387000	2.837974000
6	−1.494177000	4.605263000	1.811218000
6	−0.700865000	5.848487000	2.229709000
1	−1.621642000	3.944247000	2.675036000
1	−2.498939000	4.914338000	1.498421000
1	0.299365000	5.590748000	2.583913000
1	−1.235496000	6.354188000	3.043007000
1	−0.595005000	6.565743000	1.408690000
6	−0.660604000	4.575000000	−0.624879000
6	−0.165079000	3.787878000	−1.842036000

1	0.083803000	5.326984000	−0.357097000
1	−1.576443000	5.121933000	−0.873600000
1	0.783189000	3.282642000	−1.625785000
1	0.008113000	4.467000000	−2.684235000
1	−0.887781000	3.030933000	−2.165203000

Supplementary Table S13. DFT-optimized Cartesian coordinates for model **C** *trans* + H_2O

6	−4.258092000	1.992211000	−0.240969000
6	−3.173518000	2.872650000	−0.274109000
6	−1.969059000	2.589190000	0.380818000
6	−4.138626000	0.798339000	0.481081000
6	−1.822117000	1.345584000	1.034386000
6	−2.933135000	0.489495000	1.099320000
1	−3.300826000	3.805153000	−0.811030000
1	−2.867782000	−0.445130000	1.643048000
1	−4.952593000	0.081444000	0.543526000
6	−5.488371000	2.378340000	−0.993786000
8	−5.502249000	3.237742000	−1.856770000
8	−6.638305000	1.735054000	−0.698816000
1	−6.537725000	1.155171000	0.076218000
6	−0.936632000	3.719553000	0.492870000
6	−0.487157000	0.956743000	1.701763000
7	0.348248000	3.174592000	1.010367000
6	0.651475000	1.722598000	0.972588000
6	−0.193129000	−0.573358000	1.624699000
6	0.456528000	−1.190441000	2.872096000
1	0.443294000	−0.749378000	0.749422000
1	−1.110093000	−1.128178000	1.423574000
1	1.400303000	−0.709708000	3.141975000
1	0.660482000	−2.249985000	2.680767000
1	−0.210445000	−1.123359000	3.736171000
8	−0.533589000	1.316381000	3.090254000
1	−0.612619000	2.283940000	3.150882000
8	1.320737000	4.010552000	1.129937000
6	2.055798000	1.473903000	1.514833000
1	0.615274000	1.410801000	−0.081090000
1	2.784010000	2.055204000	0.947472000
1	2.309326000	0.417304000	1.410961000
1	2.131213000	1.758088000	2.567985000
6	−1.530349000	4.719977000	1.548448000
6	−0.721118000	5.961791000	1.943939000
1	−1.768815000	4.143696000	2.452521000
1	−2.494770000	5.043413000	1.141160000
1	0.171703000	5.707817000	2.518658000
1	−1.355847000	6.600936000	2.570504000
1	−0.416232000	6.552720000	1.074879000
6	−0.655590000	4.437513000	−0.854500000
6	−0.148868000	3.538761000	−1.986805000

1	0.082234000	5.218314000	−0.654403000
1	−1.571339000	4.950721000	−1.166207000
1	0.823503000	3.096046000	−1.743768000
1	−0.018506000	4.128794000	−2.900560000
1	−0.849995000	2.727272000	−2.209529000
8	1.999239000	4.157700000	3.885862000
1	2.859078000	4.601514000	3.964188000
1	1.864164000	4.093580000	2.919616000

Supplementary Table S14. DFT-optimized Cartesian coordinates for model **C** *trans* (carboxylate form) + H_2O

6	−4.268051000	2.000976000	−0.257390000
6	−3.186728000	2.881935000	−0.256339000
6	−1.973897000	2.586738000	0.385810000
6	−4.133492000	0.793777000	0.435229000
6	−1.822034000	1.334620000	1.021245000
6	−2.932392000	0.472475000	1.058313000
1	−3.328697000	3.828495000	−0.766022000
1	−2.866716000	−0.470755000	1.589685000
1	−4.977857000	0.112520000	0.470657000
6	−5.572148000	2.364422000	−0.975168000
8	−5.602952000	3.482076000	−1.560874000
8	−6.500398000	1.511832000	−0.921254000
6	−0.937825000	3.714453000	0.500888000
6	−0.494784000	0.948976000	1.700715000
7	0.347629000	3.169658000	1.018494000
6	0.651429000	1.718170000	0.985036000
6	−0.192563000	−0.579553000	1.630922000
6	0.451283000	−1.193898000	2.883055000
1	0.449162000	−0.757081000	0.759462000
1	−1.107764000	−1.136391000	1.427560000
1	1.392820000	−0.711813000	3.158592000
1	0.657797000	−2.253996000	2.696480000
1	−0.221195000	−1.125423000	3.742867000
8	−0.544144000	1.307770000	3.092952000
1	−0.716463000	2.262849000	3.146730000
8	1.322264000	4.004879000	1.135880000
6	2.051365000	1.472329000	1.540353000
1	0.626927000	1.406618000	−0.069155000
1	2.785303000	2.052492000	0.979008000
1	2.306789000	0.415567000	1.441765000
1	2.116769000	1.759016000	2.593566000
6	−1.522918000	4.724952000	1.550884000
6	−0.711649000	5.968631000	1.937517000
1	−1.763550000	4.156766000	2.459904000
1	−2.487541000	5.047316000	1.143908000
1	0.185388000	5.718725000	2.507633000
1	−1.342205000	6.612181000	2.564150000

1	−0.411560000	6.555143000	1.063603000
6	−0.652933000	4.427890000	−0.849059000
6	−0.146209000	3.523628000	−1.976943000
1	0.084885000	5.210417000	−0.654432000
1	−1.570150000	4.935824000	−1.164424000
1	0.826828000	3.082915000	−1.732390000
1	−0.017322000	4.107362000	−2.895229000
1	−0.847719000	2.710500000	−2.191530000
8	2.080833000	4.225337000	3.858107000
1	2.947558000	4.662414000	3.857435000
1	1.884587000	4.111693000	2.906281000

Supplementary Table S15. DFT-optimized Cartesian coordinates for structure **A**

6	−4.374184165	1.217079228	−0.722747675
6	−3.106271758	1.781922984	−0.494836354
6	−2.048826806	0.961222139	−0.134044169
6	−4.548176108	−0.172784228	−0.627908283
6	−2.228619686	−0.434897600	−0.042002583
6	−3.479722664	−1.005584421	−0.297507297
6	−0.610083006	1.345194446	0.155277498
7	−0.019284366	−0.019372993	0.390980048
6	−0.954774397	−1.065482425	0.307162017
8	1.198232832	−0.179543312	0.732966934
6	−0.420492549	2.142923853	1.472575007
6	−0.912083197	3.593069846	1.462274800
1	0.648399659	2.113460693	1.708751476
1	−0.931223167	1.590283064	2.270536819
1	−0.419946088	4.192484503	0.689714995
1	−0.691925268	4.057226047	2.430443629
1	−1.994134746	3.659680164	1.307798797
6	0.037313331	2.009234461	−1.089632933
6	1.513076895	2.407417988	−0.974193672
1	−0.561886137	2.897395330	−1.326302055
1	−0.094247316	1.322112893	−1.934238430
1	1.675717633	3.156701018	−0.191603100
1	1.846455192	2.842320141	−1.923299152
1	2.142528208	1.543016642	−0.751120004
6	−0.597838875	−2.357857910	0.542468720
6	−1.506677916	−3.551397056	0.504418179
1	−2.396642941	−3.416341570	1.132083918
1	−0.979711390	−4.440635827	0.860299786
1	−1.863030808	−3.765111965	−0.512890277
1	−2.987335184	2.853534860	−0.608942369
1	−5.507631167	−0.632540395	−0.847220733
1	−3.631680195	−2.077215178	−0.255040785
6	−5.480468936	2.143920059	−1.100398998

8	−6.750290428	1.697989055	−0.977689636
8	−5.294996435	3.275823425	−1.511266633
1	−6.786530154	0.835257339	−0.529066138
1	0.449291100	−2.517553296	0.782507655

Supplementary Table S16. DFT-optimized Cartesian coordinates for model **A** + H_2O

6	−4.400046441	1.250196411	−0.869393918
6	−3.154797760	1.867800123	−0.657679385
6	−2.090561888	1.112338939	−0.189627702
6	−4.543770242	−0.128367414	−0.647596544
6	−2.237014296	−0.273360536	0.023067151
6	−3.465954450	−0.898324565	−0.211902849
6	−0.675127674	1.561982338	0.114000133
7	−0.058362779	0.241268255	0.490542389
6	−0.957756045	−0.836354824	0.464161326
8	1.159835894	0.173124755	0.875076827
6	−0.558669641	2.476304929	1.362948984
6	−1.092464577	3.903188052	1.204813380
1	0.500402647	2.503512235	1.638869927
1	−1.083829474	1.979845267	2.187946194
1	−0.590709833	4.445848826	0.397204652
1	−0.919955435	4.458760672	2.133579160
1	−2.169653201	3.920678469	1.010542095
6	−0.000105901	2.136878218	−1.160964089
6	1.454256062	2.604041718	−1.030350634
1	−0.622437396	2.976292684	−1.494507127
1	−0.074236760	1.373806570	−1.945303880
1	1.560932068	3.416584739	−0.303203376
1	1.800440518	2.980170051	−1.999539588
1	2.110934252	1.786884539	−0.723998134
6	−0.586465351	−2.102520555	0.806775330
6	−1.482865953	−3.306326438	0.822632111
1	−2.369449499	−3.155129614	1.452065462
1	−0.943334155	−4.174043232	1.210810167
1	−1.844459286	−3.563418947	−0.182299560
1	−3.057607489	2.926803766	−0.867123722
1	−5.485553472	−0.631526207	−0.847790695
1	−3.596054639	−1.963774863	−0.070990156
6	−5.517061905	2.107728189	−1.362764022
8	−6.778951753	1.646409664	−1.218989935
8	−5.346160041	3.194980327	−1.884916868
1	−6.805542167	0.835120700	−0.681824626
1	0.448314892	−2.244308880	1.101850573
8	2.609045829	−1.925679091	2.163879755
1	2.123751054	−1.192019609	1.734463751

1	2.161276290	−2.012505658	3.020557439

Supplementary Table S17. DFT-optimized Cartesian coordinates for structure **B**

6	−4.079019541	1.613899987	−0.789305467
6	−2.830763164	2.173718889	−0.491869040
6	−1.722183211	1.385114154	−0.172962458
6	−4.210232076	0.218305270	−0.814067599
6	−1.862329235	−0.027018845	−0.181592532
6	−3.110607469	−0.579805154	−0.528453040
1	−2.748517029	3.254964902	−0.511744880
1	−5.141555189	−0.261810424	−1.101027802
1	−3.208989946	−1.655540349	−0.614684647
6	−0.381147820	2.088870713	0.144871329
7	0.643231548	1.047209882	0.461886871
6	0.606368213	−0.195973777	−0.357982005
6	−0.683569232	−0.890429976	0.064215130
8	1.137667368	0.960728015	1.642688905
6	−0.580731369	3.005282011	1.382301278
6	0.551552296	3.965572420	1.766078059
1	−0.811320682	2.356048037	2.233091363
1	−1.480194205	3.598681660	1.191318716
1	1.486701305	3.432966073	1.952539446
1	0.273102546	4.494241837	2.685509910
1	0.725705422	4.724046229	0.996219329
6	0.074766195	2.874550324	−1.136517044
6	1.581504324	3.116411958	−1.299840545
1	−0.456653568	3.833163284	−1.131978549
1	−0.284816855	2.340093555	−2.023040975
1	2.020292569	3.649250706	−0.452901786
1	1.753306902	3.717857470	−2.200261780
1	2.128697449	2.175887063	−1.421882134
6	−0.687433734	−2.096258370	0.663853685
6	−1.832768154	−2.835134765	1.298532888
1	−2.682668838	−2.188578982	1.532000002
1	−1.492529748	−3.306605502	2.228059818
1	−2.194175010	−3.647410518	0.651873912
1	0.268265462	−2.609508317	0.739207890
1	0.502309470	0.135882781	−1.397841071
6	1.907921683	−0.973809456	−0.218040142
1	1.879701290	−1.864194087	−0.852120594
1	2.083946038	−1.276691877	0.815657717
1	2.746626922	−0.348999303	−0.541417806
6	−5.199279602	2.544072064	−1.109739891
8	−6.458087352	2.052294465	−1.068531923
8	−5.039269538	3.715569954	−1.404384257

1	−6.477451436	1.148561997	−0.708132283

Supplementary Table S18. DFT-optimized Cartesian coordinates for model **B** + H_2O

6	−4.037676122	1.622891531	−0.862747936
6	−2.780154479	2.168143091	−0.582303993
6	−1.683321546	1.366605674	−0.253291655
6	−4.195288000	0.229604781	−0.850940764
6	−1.854236553	−0.037905193	−0.212662919
6	−3.111673355	−0.580577278	−0.540234573
1	−2.680722916	3.247455175	−0.616408785
1	−5.135676843	−0.240125826	−1.125048096
1	−3.229831204	−1.656757487	−0.588843130
6	−0.344764303	2.061616050	0.071510888
7	0.707230676	1.025559481	0.243237049
6	0.600813248	−0.281360342	−0.461692148
6	−0.689239134	−0.908362778	0.062400083
8	1.681039361	1.236543309	1.053192110
6	−0.524160662	2.829518166	1.422429384
6	0.453496548	3.954464464	1.781313625
1	−0.523653406	2.075075552	2.218400971
1	−1.531689733	3.257228054	1.404450261
1	1.481708415	3.595815868	1.844471870
1	0.171376288	4.365129185	2.758081502
1	0.409624855	4.777453779	1.059797813
6	0.050440346	2.978114031	−1.141032297
6	1.535307875	3.331942764	−1.290835150
1	−0.541746741	3.897504430	−1.067899358
1	−0.276616756	2.477069941	−2.059693267
1	1.930710213	3.880016311	−0.433052050
1	1.662187155	3.960057951	−2.180244654
1	2.148770922	2.435690944	−1.427826659
6	−0.709505648	−2.083573498	0.722894212
6	−1.867910326	−2.762670195	1.400858440
1	−2.700889808	−2.085606478	1.605601552
1	−1.533840992	−3.198274875	2.349638201
1	−2.248988545	−3.595384600	0.793157551
1	0.231069213	−2.624132009	0.800395359
1	0.458963865	−0.046481436	−1.526249072
6	1.881986438	−1.088090517	−0.303135231
1	1.794308890	−2.017280002	−0.872297391
1	2.082906097	−1.327980001	0.743425284
1	2.730594000	−0.521279159	−0.694271015
6	−5.142813689	2.565758699	−1.202910749
8	−6.409410124	2.098043108	−1.146267600
8	−4.961058152	3.726047828	−1.526607264

1	−6.444802258	1.203218244	−0.765608607
8	0.908426009	−0.380085095	3.246356019
1	1.310119704	0.233495515	2.598651856
1	0.167831178	−0.761457158	2.747850335

Supplementary Table S19. DFT-optimized Cartesian coordinates for structure **C** *cis*

6	−4.119160656	2.946629402	−1.195913054
6	−3.067634165	3.799780960	−0.851862249
6	−1.813568165	3.314812096	−0.458390697
6	−3.913881733	1.561146650	−1.149136875
6	−1.594325501	1.920442216	−0.474449704
6	−2.664180283	1.068781939	−0.795414509
1	−3.258878561	4.866560048	−0.879384767
1	−4.695722678	0.856066034	−1.417116020
1	−2.519854653	−0.005253055	−0.773108972
6	−5.411336580	3.570704254	−1.607908491
8	−6.516962297	2.794503890	−1.600877340
8	−5.515309035	4.735816534	−1.947467044
6	−0.773202721	4.342911569	0.036233632
7	0.493907029	3.624698891	0.332875735
6	0.878040007	2.373045851	−0.355121867
6	−0.235917016	1.336334123	−0.077631672
8	1.410121110	4.226928914	1.006795281
6	−1.336029778	4.931928567	1.376791961
6	−0.708290666	6.196320196	1.975765453
1	−1.304952713	4.122770056	2.118083918
1	−2.397250002	5.137190057	1.204146321
1	0.355472157	6.065140931	2.178208406
1	−1.216595421	6.426182349	2.919764682
1	−0.838934296	7.062805186	1.319192574
6	−0.557785610	5.432977570	−1.072144125
6	0.732093152	6.262863352	−1.037895159
1	−1.417379022	6.112450806	−1.025586072
1	−0.619798667	4.936878762	−2.046947731
1	1.622897558	5.635928610	−1.131180464
1	0.836610970	6.851789951	−0.124967078
1	0.722397573	6.956407776	−1.887391518
8	−0.253395060	1.086561082	1.348266629
6	0.080341729	−0.064378766	−0.656960241
6	−0.063032878	−0.332531980	−2.162371594
1	−0.563308747	−0.764256977	−0.111547341
1	1.103214215	−0.302276785	−0.340005452
1	−1.041330148	−0.032893704	−2.549206762
1	0.044601441	−1.408723266	−2.338817705
1	0.698668887	0.171365708	−2.759707073

6	1.267587540	2.625333642	−1.818894384
1	1.763236799	2.043264717	0.193863757
1	0.399697712	2.790395284	−2.464451924
1	1.833768916	1.777283245	−2.209761052
1	1.916352444	3.504724108	−1.873408010
1	−6.340050649	1.921323882	−1.209373755
1	−0.268521537	1.948115323	1.800502352

Supplementary Table S20. DFT-optimized Cartesian coordinates for model **C** *cis* + H_2O

6	−4.090101980	2.883713470	−0.976745449
6	−3.039961547	3.788168002	−0.804999220
6	−1.742786057	3.369579550	−0.477069235
6	−3.839910060	1.513405443	−0.815607988
6	−1.478386740	1.987103129	−0.402244892
6	−2.548904149	1.086006440	−0.538232332
1	−3.266236361	4.843234196	−0.912020869
1	−4.621152224	0.768775651	−0.939936856
1	−2.365915845	0.022521935	−0.431801139
6	−5.429946510	3.437929466	−1.330697605
8	−6.508437936	2.643388505	−1.155324610
8	−5.596798963	4.563169375	−1.766635454
6	−0.707897465	4.460866698	−0.135177729
7	0.593016816	3.801452191	0.141265479
6	0.994033634	2.513582383	−0.463162408
6	−0.073924200	1.443483885	−0.118091993
8	1.496018996	4.453300041	0.791451316
6	−1.210337764	5.148723753	1.184076284
6	−0.625428009	6.500742883	1.611703999
1	−1.080424251	4.421844112	1.993529503
1	−2.290206778	5.281439198	1.068081806
1	0.454763163	6.452190043	1.758296226
1	−1.090267232	6.795819133	2.559980358
1	−0.847991379	7.288630402	0.884562556
6	−0.580503283	5.459942247	−1.338454663
6	0.686632645	6.319976356	−1.432314585
1	−1.455602303	6.119805382	−1.299871605
1	−0.676914527	4.885264642	−2.266277148
1	1.588736569	5.709509013	−1.525197257
1	0.819686774	6.977429741	−0.571213907
1	0.616752928	6.947903509	−2.328313121
8	0.022707461	1.121834086	1.279601398
6	0.232836773	0.080698989	−0.800539349
6	−0.130089166	−0.159090635	−2.273971485
1	−0.276990743	−0.672760174	−0.189272647
1	1.304708345	−0.102561939	−0.652137711

1	−1.194033998	0.002474174	−2.470442564
1	0.094676947	−1.202299700	−2.524556222
1	0.433500887	0.468947842	−2.965947255
6	1.367678836	2.696934170	−1.942052917
1	1.897344104	2.241384534	0.088492866
1	0.495902113	2.859527277	−2.581900343
1	1.906969597	1.818955345	−2.304324968
1	2.036924282	3.556941824	−2.038486009
1	−6.274548981	1.814853414	−0.701695521
1	0.184197312	1.924303607	1.830578372
8	0.828025677	3.064565284	3.122911508
1	1.553190919	2.540380114	3.502639300
1	1.273803672	3.682261012	2.506096089

Supplementary Table S21. DFT-optimized Cartesian coordinates for structure **D**

6	−5.117478434	2.167564253	−0.778028230
6	−3.922105955	2.814358305	−0.452379116
6	−2.757882475	2.099367704	−0.138194931
6	−5.163704887	0.764925123	−0.797597328
6	−2.813894971	0.696781490	−0.157507495
6	−4.015676759	0.049020145	−0.489515826
6	−1.476612657	2.887804941	0.190948963
7	−0.322710159	1.953071164	0.395060907
6	−0.318403846	0.614368500	−0.059937952
6	−1.605678998	−0.137555809	0.202074822
8	0.754316663	2.460504507	0.887403287
6	−1.727775586	3.669267499	1.523937941
6	−0.833591143	4.860029260	1.887083382
1	−1.704013166	2.930199352	2.335029072
1	−2.759462275	4.032749509	1.480645667
1	0.215502674	4.573378349	1.965658144
1	−1.158828892	5.261162428	2.854583688
1	−0.922121098	5.670460605	1.155364001
6	−1.177258674	3.814513677	−1.041924046
6	0.238267377	4.370209752	−1.241204872
1	−1.883634769	4.651368439	−0.982785158
1	−1.447240497	3.252471939	−1.943768038
1	0.599613722	4.942154078	−0.385314349
1	0.226642053	5.034409310	−2.114461302
1	0.959780240	3.573820004	−1.442156340
1	−3.923556733	3.898953410	−0.450068215
1	−6.081386064	0.248045940	−1.054330381
1	−4.038931623	−1.037984731	−0.505872941
6	−1.683586123	−0.602622964	1.679245530
1	−2.615341807	−1.150470149	1.859005624

1	−1.653742085	0.253116750	2.362890186
1	−0.840949195	−1.262456039	1.912224306
1	−1.607369872	−1.033210187	−0.427164513
6	0.771165285	0.005065597	−0.575949171
6	2.127920776	0.566846296	−0.879136281
1	2.458639546	0.226888337	−1.868410624
1	2.870465661	0.198239779	−0.156773409
1	2.149897298	1.656566489	−0.844472441
1	0.639581053	−1.050213348	−0.810161529
6	−6.301005056	3.006513832	−1.097240508
8	−7.400487464	2.282694008	−1.391993487
8	−6.306147199	4.228078850	−1.099631241
1	−8.120265885	2.913717607	−1.588178794

Supplementary Table S22. DFT-optimized Cartesian coordinates for model **D** + H_2O

6	−5.198407360	2.208947547	−0.671305982
6	−3.973499205	2.809598930	−0.368948860
6	−2.818336056	2.051199302	−0.132416893
6	−5.282638102	0.810263877	−0.751714148
6	−2.908958525	0.653199621	−0.222609105
6	−4.141721659	0.052824591	−0.530124174
6	−1.498579573	2.794158783	0.147842808
7	−0.393746109	1.809605425	0.390599356
6	−0.402669335	0.501314936	−0.145748233
6	−1.713267234	−0.236266199	0.035412148
8	0.658182324	2.233332710	1.004962259
6	−1.674167997	3.658191058	1.440582640
6	−0.787655578	4.888820970	1.663903810
1	−1.577387547	2.976534058	2.293795397
1	−2.712192389	4.002317891	1.449909742
1	0.273324315	4.641791088	1.720000703
1	−1.079460071	5.359058699	2.611088805
1	−0.929036189	5.637529031	0.876635610
6	−1.172881723	3.617777768	−1.150713351
6	0.264559211	4.091915760	−1.389200044
1	−1.847820360	4.481882441	−1.154222544
1	−1.468180941	2.999136685	−2.006105782
1	0.645564146	4.737730272	−0.597118315
1	0.292260610	4.659928266	−2.326884823
1	0.951126761	3.247464532	−1.500997137
1	−3.942260660	3.892820335	−0.325461704
1	−6.223893835	0.328316809	−0.990146884
1	−4.194983964	−1.030986094	−0.597869951
6	−1.793703639	−0.848977549	1.458353516
1	−2.732373793	−1.399494293	1.585048948

1	−1.751461512	−0.065510420	2.223633033
1	−0.959632105	−1.539593220	1.621269186
1	−1.739156486	−1.062202891	−0.683294014
6	0.690555494	−0.105842275	−0.656416676
6	2.081846321	0.413034620	−0.859776975
1	2.388654934	0.259049018	−1.902530028
1	2.789148491	−0.157417798	−0.241863324
1	2.193045889	1.465353544	−0.601649885
1	0.532481204	−1.135317409	−0.974767482
6	−6.372007270	3.090398204	−0.901144693
8	−7.501633990	2.407014974	−1.177252503
8	−6.345177560	4.310548085	−0.849465759
1	−8.213475527	3.062824037	−1.311307455
8	2.791612153	4.101336550	0.939788908
1	3.060896011	3.953331398	1.860467788
1	2.012638431	3.522886331	0.836752066

Supplementary Table S23. DFT-optimized Cartesian coordinates for structure **E**

6	−4.353965660	1.847894055	0.856043439
6	−3.135648771	2.502532392	0.629723876
6	−2.030897523	1.848938681	0.077935909
6	−4.457991991	0.488820255	0.546861785
6	−2.135723716	0.467122915	−0.209785593
6	−3.352464947	−0.183824026	0.033546893
6	−0.765629897	2.656476915	−0.247539557
7	0.392490169	1.720092038	−0.458762932
6	0.334137492	0.294251737	−0.014247030
6	−0.917973500	−0.304382326	−0.715479547
6	−0.967735986	3.413177984	−1.599995052
6	−2.016212960	4.528242658	−1.623920244
1	−1.218659292	2.663085346	−2.360151250
1	0.001378263	3.825764181	−1.885485490
1	−3.027583021	4.159061270	−1.429260517
1	−2.022033337	4.989594991	−2.618071588
1	−1.796341700	5.319406024	−0.898434535
6	−0.389217005	3.606299580	0.929881221
6	0.887561839	4.434216440	0.746831118
1	−0.301033363	2.986361499	1.829334479
1	−1.220995836	4.294018808	1.108848512
1	1.767423511	3.802796648	0.596283650
1	1.055488113	5.044033445	1.642199881
1	0.814474343	5.115872744	−0.107956473
8	1.355300885	2.098388609	−1.220353479
8	0.080451461	0.249118396	1.380956861
6	1.650318445	−0.417752459	−0.369426175

6	2.892991004	0.077594433	0.384340550
1	1.811759082	−0.346486378	−1.448658079
1	1.506282992	−1.474978171	−0.131811259
1	3.091567221	1.133301995	0.186910658
1	3.766315239	−0.500352722	0.063966755
1	2.800928425	−0.067943463	1.468196231
6	−1.042692456	−1.828396814	−0.573066856
1	−1.907155050	−2.198825379	−1.130675650
1	−1.152466288	−2.126730897	0.474345508
1	−0.164868897	−2.328749277	−0.987036857
1	−0.785357897	−0.083115353	−1.784240599
1	−3.082747952	3.549260649	0.898627104
1	−5.366787274	−0.077364878	0.733945828
1	−3.448959386	−1.243667458	−0.167554016
6	−5.466007968	2.645930660	1.446848558
8	−6.710885589	2.120095942	1.413634946
8	−5.311466491	3.742664952	1.955864277
1	−6.737904263	1.292575779	0.902752726
1	0.882153533	0.546213578	1.843695012

Supplementary Table S24. DFT-optimized Cartesian coordinates for model **E** + H_2O

6	−4.321586554	0.557530335	−0.737087491
6	−3.194264977	1.357551178	−0.526559496
6	−1.999691373	0.834471901	−0.020862097
6	−4.253321309	−0.807400407	−0.435361105
6	−1.917861183	−0.550536133	0.248908257
6	−3.061258135	−1.341057129	0.041227601
6	−0.833327402	1.816227429	0.219846081
1	−3.271838922	2.411294601	−0.770516624
1	−5.115451931	−1.448426696	−0.582043694
1	−3.024404371	−2.402982809	0.251719744
7	0.342555521	1.034859243	0.726613275
6	0.579918866	−0.357417062	0.213562683
6	−0.617444576	−1.190929772	0.741836169
8	0.843330183	1.334194284	1.874037200
6	−5.549426905	1.198042723	−1.272123027
8	−6.575944929	0.335697615	−1.423054678
1	−7.332076833	0.842658449	−1.777981108
8	−5.647470695	2.382658292	−1.554699266
6	−1.284415065	2.820793080	1.323588728
6	−0.427756811	4.063949319	1.590394054
1	−1.408628969	2.255472576	2.252992966
1	−2.284116921	3.158948025	1.034226831
1	0.594918989	3.803818718	1.869976201
1	−0.873191658	4.627763953	2.419006952

1	−0.396163802	4.733119890	0.724087031
6	−0.474234571	2.535053329	−1.126857919
6	0.959183149	3.062193898	−1.268816875
1	−1.180238002	3.365305976	−1.245496346
1	−0.668048818	1.839999729	−1.947707146
1	1.688535342	2.245959690	−1.272043196
1	1.240781961	3.753433778	−0.471485444
1	1.056350558	3.592591874	−2.223370797
6	−0.496196950	−2.685028509	0.398029736
1	−0.572186236	−1.097204110	1.835949644
1	−1.296868511	−3.261000380	0.868481373
1	0.444969593	−3.092669993	0.774182581
1	−0.538778210	−2.848770410	−0.683195128
6	1.951330498	−0.865427010	0.693264081
6	3.148218057	0.009527667	0.298247835
1	2.080779224	−1.857679492	0.251362823
1	1.927338788	−1.000269992	1.777121683
1	3.235972605	0.128893855	−0.789432402
1	4.075073912	−0.465927696	0.636283646
1	3.089112877	1.001302090	0.751901535
8	0.490908432	−0.349561091	−1.194863407
1	1.317056904	0.011448007	−1.557971636
8	0.535427666	−0.417315513	4.079669016
1	0.708386238	0.174478620	3.319604286
1	−0.382204744	−0.215925920	4.323126870

[1] C.D. Smith, J.P. Bartley, S.E. Bottle, A.S. Micallef, D.A. Reid, Electrospray ionization mass spectrometry of stable nitroxide free radicals and two isoindoline nitroxide dimers, J. Mass Spectrom. 35 (2000) 607–611. https://doi.org/10.1002/(SICI)1096-9888(200005)35:5<607::AID-JMS967>3.0.CO;2-7.
